# In silico, in vitro and in vivo characterization of host-associated *Latilactobacillus curvatus* strains for potential probiotic applications in farmed Atlantic salmon (*Salmo salar*)

**DOI:** 10.1038/s41598-022-23009-y

**Published:** 2022-11-01

**Authors:** Hannah S. Cathers, Shrinivasrao P. Mane, Nilesh R. Tawari, Jayanth Balakuntla, Germán Plata, Madan Krishnamurthy, Alicia MacDonald, Marilyn Wolter, Niel Baxter, Julian Briones, Akshitha Nagireddy, Gregory Millman, Roberto E. Martin, Arvind Kumar (Mahajan), Dharanesh Gangaiah

**Affiliations:** 1 Elanco Animal Health, 2500 Innovation Way, Greenfield, IN 46140 USA; 2 Elanco Innovation and Alliance Centre, 22, 3Rd Cross Rd, SR Layout, Murgesh Pallya, Bengaluru, Karnataka India; 3 Elanco Animal Health, 37 McCarville St, Charlottetown, PEI Canada; 4 Elanco Animal Health, Inc., Ruta 5 Sur Km 1012, Puerto Varas, Los Lagos, Chile; 5 BiomEdit, LLC, 10100 Lantern Road, Fishers, IN 46037 USA

**Keywords:** Microbiome, Bacteriology, Symbiosis

## Abstract

Salmon aquaculture is the fastest growing animal protein production system in the world; however, intensive farming leads to poor weight gain, stress, and disease outbreaks. Probiotics offer the potential to enhance growth performance and feed efficiency in Atlantic salmon, as well as immunostimulate fish against common pathogens, benefitting farmers and consumers with more efficient production. Here, we isolated and identified 900 native microbial isolates including 18 *Lactobacilli* from the farmed salmon intestines. Based on whole-genome sequencing and phylogenetic analysis, the *Lactobacillus* candidates belonged to *Latilactobacillus curvatus* (*L. curvatus*) species and formed two distinct phylogenetic groups. Using bioinformatics and in vitro analyses, we selected two candidates *L. curvatus* ATCC PTA-127116 and *L. curvatus* ATCC PTA-127117, which showed desirable safety and probiotic properties. The two *L. curvatus* candidates were evaluated for safety and efficacy (higher final weight) in Atlantic salmon alongside spore-forming *Bacilli* isolated from salmon, poultry, and swine. All the tested candidates were safe to salmon with no adverse effects. While we did not see efficacy in any *Bacillus* supplemented groups, compared to untreated group, the group administered with the two *L. curvatus* strains consortium in feed for seven weeks in freshwater showed indicators of improvement in final body weight by 4.2%. Similarly, the two *L. curvatus* candidates were also evaluated for safety and efficacy in Atlantic salmon in saltwater; the group administered with the two *L. curvatus* strains consortium in feed for 11 weeks showed indicators of improvement in final body weight by 4.7%. Comprehensive metabolomics analyses in the presence of different prebiotics and/or additives identified galactooligosaccharide as a potential prebiotic to enhance the efficacy of two *L. curvatus* candidates. All together, these data provide comprehensive genomic, phenotypic and metabolomic evidence of safety and desirable probiotic properties as well as indicators of in vivo efficacy of two novel endogenous *L. curvatus* candidates for potential probiotic applications in Atlantic salmon. The in vivo findings need to be confirmed in larger performance studies, including field trials.

## Introduction

Salmon is a valuable protein source worldwide; since 2016, it has been the second-most popular seafood consumed in the United States^1^. Salmon farming is critical to fill this demand as aquaculture provides 70% of global salmon production^2^. Atlantic salmon are attractive to farmers as prices and profit margins are high due to strong demand, and require significantly less fresh water, space, and feed to produce the same mass of protein than terrestrial agriculture^3^. The bulk of the salmon production cycle takes place in non-potable saltwater^4^. Atlantic salmon meat is also attractive to consumers for its nutritional value including omega-3 fatty acids^5–7^. However, the Atlantic salmon production cycle is relatively long at three years, which can make salmon farming capital-intensive and volatile^8^ and salmon feed is the largest cost component involved in salmon production^8^. In an effort to improve feed efficiency, plant and insect proteins have been integrated into salmon feed^9–11^. This can result in gut inflammation and poor weight gain due to antinutrients^10^ and changes in the fatty acid profile of salmon meat, specifically decreasing desirable omega-3 long-chain fatty acids^12^. Salmon farming is also impacted by losses from diseases like sea lice^13^.

Probiotics are a promising approach to improve salmon weight gain and disease resistance, offering potential solutions for the two major challenges in aquaculture. *Lactobacillus*, first bacteriologically described in 1901^14^, is a popular probiotic candidate genus of lactic acid bacteria with a long history of safe use^15,16^, and many studies have shown their efficacy in modulating terrestrial host immune systems^17–20^. They dominate the intestine of healthy fish^21,22^ and favorably modulate fish gut microbiome^22^, suggesting that they may be adapted for survival in the fish gut environment. *Lactobacilli* have been repeatedly demonstrated to improve fish disease resistance via immunostimulation^17–20^. This effect likely stems from a combination of mechanisms such as humoral immune modulation^21,22^, bacteriocin production^23^, and lymphocyte modulation^24,25^. Lactic acid bacteria can directly inhibit aquatic pathogens like *Aeromonas*^24–27^, and when combined with prebiotics, form a synbiotic which can also improve humoral immune response and weight gain^28^. More specifically, *Latilactobacillus curvatus* (*L. curvatus*) strains were shown to inhibit the adhesion of fish and shrimp pathogens via producing antagonistic compounds^29,30^, and possess desirable safety and technological properties for use as potential probiotics for Argentinean anchovies^31^. Administration of *L. curvatus* strains isolated from the gastrointestinal tract of beluga fish (*Huso huso*) improved growth, survival, and digestive enzyme activity of beluga fry^32^.

Various terrestrial and aquatic sources can yield probiotics for use in aquaculture, including cheese^33^, humans, crops, soil^34,35^ or wild fish specimens^22,36^. Native microbial species are already adapted to the temperature, pH, osmotic pressure, and native antimicrobial activity seen in farmed fish^26^. While terrestrial probiotic candidates may be able to survive under these conditions, native species may already be optimized to conferring probiotic benefits^37^, and colonization and positive effects may last longer^38^.

Here, we present screening, identification, and analysis of native *Latilactobacillus* candidates, recently differentiated within *Lactobacillus*^39^ for probiotic use in salmon. Our study includes whole genome sequencing feature analysis, as well as extensive metabolomics analysis in the presence of several prebiotic candidates towards the design of a synbiotic. Results from a clinical study show indicators of improvement in salmon parr growth performance. These data and analyses will guide the design of future larger studies to develop a probiotic product towards a safe, sustainable, and effective performance improvement in Atlantic salmon.

## Methods

### Probiotic candidate isolation

Probiotic candidates were isolated from healthy salmon samples received from Chile, Norway, and North America over a seven-month period. On each site, selected stock fish were humanely euthanized according to the farm’s standard husbandry procedures e.g. overdose of an approved fish anesthetic, before packaging whole or processing for tissues prior to cold chain shipment. The fish were not exposed to any commercial probiotics. On site or upon receipt at Elanco US in Greenfield IN, fish whole gut, skin and gill samples were excised, and the gut samples were separated aseptically into foregut and hindgut. Each sample was homogenized completely by hand in Whirl–Pak bags (Whirl–Pak; Madison, WI) in De Man Rogosa and Sharpe broth (MRS) (Becton Dickinson (BD); Franklin Lakes, NJ). Aliquots were heat-treated at 100 °C for 10 min to select for spore formers, targeting *Bacillus* spp. Dilutions were prepared to 10^–2^ in PBS (Gibco Thermo Fisher; Hampton, NH) and 0.1 mL of each dilution was spread over the surface of plates of MRS agar (BD) supplemented with amphotericin B (Thermo Fisher) for lactic acid bacteria, and Luria–Bertani (LB) agar (BD) for *Bacillus* species. LB plates were incubated aerobically at 15 °C for three days, and MRS plates were incubated at 15 °C or 23 °C under microaerophilic conditions in a GasPak EZ Campy Container system (BD) for 4 days before colonies were picked and re-isolated on fresh medium three times. Lactic acid bacteria were passaged under both aerobic and microaerophilic conditions at 15 °C and 23 °C. Three of the candidates used in salmon studies were *Bacillus* isolated in the same way from chicken cecum and swine intestine described previously^40,41^.

### Bacterial identification

Probiotic candidate strains were identified using 16S rRNA gene sequencing. Briefly, lactic acid bacterial strains were grown on *Lactobacilli* MRS agar for 36–48 h under microaerophilic conditions at 25 °C using BD GasPak container and sachets (BD). *Bacillus* strains were grown on LB agar for 36–48 h under aerobic conditions at 25 °C. Patched colonies were resuspended in 50 µL of nuclease-free water and heated at 100 °C for 10 min. The debris were pelleted by brief centrifugation and the supernatant was used as a template for PCR. Sanger sequencing was sent to TacGen for analysis (TacGen; Richmond, CA) using U16Sf 5’- AGAGTTTGATCCTGGCTCAG-3’ and U16Sr R, 5'-CTTGTGCGGGCCCCCGTCAATTC-3'. The sequences were then searched against the NCBI nucleotide collection (nr/nt) database using the BLAST algorithm^42^.

Selected isolates were further identified with colony PCR using universal bacterial primer U16Sf and U16Sr in a 25 µL master mix consisting of 12.5 µL NEB Phusion master mix (NEB) and 2.5 µL 10 µM primer mix. These PCR products were submitted to an outside partner (ACGT; Wheeling, IL) for sequencing using U16Sr, and identified using BLAST analysis against NCBI 16S rRNA species database^42^.

### Isolation of genomic DNA

Genomic DNA for Illumina sequencing was isolated using the DNeasy Blood and Tissue kit (Qiagen; Hilden, Germany) for Gram-positive bacteria. Briefly, *Lactobacillus* strains were grown in MRS broth overnight under aerobic conditions for 14–16 h without shaking. The cells were pelleted by centrifugation at 4000×*g* for 10 min at 4 °C. The pellet was washed once in 1 mL of phosphate buffered saline (PBS, Invitrogen) and resuspended in 0.2 mL P1 buffer containing 100 µg/mL of RNase (Qiagen) and 6.25 mg/mL of lysozyme (Sigma Aldrich) and incubated at 37 °C overnight. After incubation, 20 µL of proteinase K (Qiagen) was added, mixed several times, and incubated at 55 °C for 1 h. Subsequently, DNA was purified following supplier protocol, except that the DNA was eluted in 100 µL of distilled H_2_O. Isolated DNA was quantified using Qubit 3.0 (Invitrogen) and DNA integrity was confirmed by agarose gel electrophoresis.

### Whole-genome sequencing (WGS) and assembly

Whole-genome sequencing (WGS) of *Lactobacillus* isolates was performed using the Illumina platform. Library preparations were performed according to the manufacturer’s instructions for the Nextera DNA Flex Library Prep kit (Illumina; San Diego, CA). The concentration of DNA was confirmed using HS DNA Assay kit with the Qubit 3.0 (Invitrogen). Approximately, 300 ng of genomic DNA was subjected to tagmentation process by enzymatic fragmentation, followed by addition of sequence-specific overhangs using Bead-Linked Transposome technology. Following tagmentation, the samples were amplified with 5 cycles of PCR, using index labelled primers specific to the inserted sequences. Fragments were separated by size exclusion using SPRI-beads to obtain fragment sizes of ~ 600 base pairs. The eluted libraries were then confirmed for size and quality using the 4200 TapeStation High Sensitivity D1000 reagents (Agilent Technologies; Santa Clara, CA) and concentrations determined using Qubit HS DNA Assay kit (Invitrogen). Each library was diluted to a 4 nM stock and 5 µL of each library was combined into a pooled library. The pooled library was then denatured by incubating with 0.2 N NaOH at room temperature for 5 min and diluted to a final concentration of 12 pM. The diluted pooled libraries were then added to the reagent cartridge (MiSeq Reagent Kit v3, Illumina) and sequenced using MiSeq. Low quality reads trimming, and adaptor removal was performed using Trimmomatic software version 0.39^43^. Paired end reads were filtered using leading, trailing window of 20 and sliding window of 5 with average quality score of 20 to retain high quality reads. High-quality reads were used for de novo genome assembly with Unicycler^44^ using the default assembly method. Scaffolds were filtered for a minimum of 200-bp read length. The quality of the subsequent assemblies was assessed by mapping the reads using Burrows-Wheeler Aligner^45^. Genome completeness was found to be 99.46% for all the strains, using the CheckM lineage^46^.

The genomes of *Latilactobacillus curvatus* (*L. curvatus*) strains PTA-127116 and PTA-127117 were further sequenced using PacBio platform. Bacterial pellet samples were sent to DNA Link, Inc (San Diego, CA) for WGS using PacBio RSII platform (PacBio; Menlo Park, CA). Briefly, 20 kb DNA fragments were generated by shearing genomic DNA using the Covaris G-tube according to the manufacturer’s recommended protocol (Covaris; Woburn, MA). Smaller fragments were purified by the AMpureXP bead purification system (Beckman Coulter; Brea, CA). For library preparation, 5 µg of genomic DNA was used. The SMRTbell library was constructed using SMRTbell™ Template Prep kit 1.0 (PacBio®). Small fragments were removed using the BluePippin Size selection system (Sage Science; Beverly, MA). The remaining DNA sample was used for large-insert library preparation. A sequencing primer was annealed to the SMRTbell template and DNA polymerase was bound to the complex using DNA/Polymerase Binding kit P6 (PacBio®). Following the polymerase binding reaction, the MagBead was bound to the library complex with MagBeads kit (PacBio®). This polymerase-SMRTbell-adaptor complex was loaded into zero-mode waveguides. The SMRTbell library was sequenced by 2 PacBio® SMRT cells (PacBio®) using the DNA sequencing kit 4.0 with C4 chemistry (PacBio®). A 1 × 240-min movie was captured for each SMRT cell using the PacBio® RS sequencing platform. The reads were assembled using HGAP.3 protocol by DNA link, Inc.

### Genome annotation, and feature prediction

Genome annotation was carried out using NCBI Prokaryotic Genome Annotation Pipeline (PGAP), which combines alignment-based methods with methods of predicting protein-coding and RNA genes and other functional elements directly from the sequence^47^. The biosynthetic gene clusters for secondary metabolites were predicted using Antismash 5.0^48^.

### Data deposition

The raw sequencing reads, genome assemblies and annotations were deposited under the genome and bioproject accession numbers listed in Table 1.

**Table 1:** Accession numbers for *Latilactobacillus* strains.

Sample name	Organism	Isolate	Biosample accession	Genome accession	Bioproject accession
ATCC PTA-127116	*L. curvatus*	ELA204093	SAMN21465945	JAIULW000000000	PRJNA762592
ATCC PTA-127117	*L. curvatus*	ELA204100	SAMN21465946	JAIULV000000000	PRJNA762592
LcELA388	*L. curvatus*	ELA214388	SAMN21465947	JAIULU000000000	PRJNA762592
LsELA391	*L. sakei*	ELA214391	SAMN21465948	JAIULX000000000	PRJNA762593
LcELA2	*L. curvatus*	ELA214002	SAMN23139425	JAJJOL000000000	PRJNA762592
LcELA23	*L. curvatus*	ELA204023	SAMN23139426	JAJJOM000000000	PRJNA762592
LcELA29	*L. curvatus*	ELA204029	SAMN23139427	JAJJON000000000	PRJNA762592
LcELA33	*L. curvatus*	ELA204033	SAMN23139428	JAJJOO000000000	PRJNA762592
LcELA59	*L. curvatus*	ELA214059	SAMN23139429	JAJJOP000000000	PRJNA762592
LcELA60	*L. curvatus*	ELA214060	SAMN23139430	JAJJOQ000000000	PRJNA762592
LcELA61	*L. curvatus*	ELA214061	SAMN23139431	JAJJOR000000000	PRJNA762592
LcELA62	*L. curvatus*	ELA214062	SAMN23139432	JAJJOS000000000	PRJNA762592
LcELA92	*L. curvatus*	ELA204092	SAMN23139433	JAJJOT000000000	PRJNA762592
LcELA96	*L. curvatus*	ELA204096	SAMN23139434	JAJJOU000000000	PRJNA762592
LcELA98	*L. curvatus*	ELA204098	SAMN23139435	JAJJOV000000000	PRJNA762592
LfELA68	*L. fuchuensis*	ELA214068	SAMN23139436	JAJJOH000000000	PRJNA780402
LsELA64	*L. sakei*	ELA214064	SAMN23139437	JAJJOI000000000	PRJNA762593
LsELA65	*L. sakei*	ELA214065	SAMN23139438	JAJJOJ000000000	PRJNA762593

### Phylogenetic analyses

Phylogenetic relationships of the genomes were explored with UBCG v3.0 using default settings^49^. This software tool employs a set of 92 single-copy core genes commonly present in all bacterial genomes. These genes were then aligned and concatenated within UBCG using default parameters. The estimation of robustness of the nodes is done through the gene support index (GSI), defined as the number of individual gene trees, out of the total genes used, that present the same node. A maximum-likelihood phylogenetic tree was inferred using FastTree v.2.1.10 with the GTR + CAT model^50^.

### Identification of prophages, transposases, and other insertion sequences

Insertion sequence (IS) prediction was completed using ISEscan v.1.7.2.1^51^. Prophage prediction was done using PhiSpy v4.2.6, which combines similarity‐ and composition‐based strategies^52^.

### Antimicrobial susceptibility profiling

Candidates were sent to Microbial Research Inc (Fort Collins, CO) for antimicrobial susceptibility analysis, performed as previously described^40,41^ for *Bacillus. Latilactobacillus* strains were also analyzed at Microbial Research Inc using broth microdilution method in laked horse blood (LHB) medium [Mueller Hinton broth (BD) containing 5% horse blood] following Clinical and Laboratory Standards Institute (CLSI) guidelines. Two-fold dilutions of the clinically relevant antibiotics (Clindamycin, Chloramphenicol, Erythromycin, Gentamicin, Kanamycin, Streptomycin, Tetracycline and Ampicillin; Sigma Aldrich; St. Louis, MO) were prepared in LHB medium. Approximately 50 µL of 1 × 10^5^ CFUs/mL of the *Latilactobacillus* cells were added into each well. “No antibiotic” and “medium” alone controls were included. *Escherichia coli* ATCC 25923, *Pseudomonas aeruginosa* ATCC 27853, *Staphylococcus aureus* ATCC 29213, *Enterococcus faecalis* ATCC 29212, and *Streptococcus pneumonia* ATCC 49619 were used as quality control organisms. The *Latilactobacillus* plates were incubated for 24–48 h under microaerophilic conditions and *Bacillus* plates were incubated aerobically. Minimum inhibitory concentration (MIC) was defined as the lowest concentration of antibiotic that showed complete inhibition of candidate growth. The strains were classified as susceptible or resistant using the microbiological cut offs established by European Food Safety Agency (EFSA)^53^.

### Preparation of probiotics, premixes, and test products (TPs)

*Latilactobacillus* spp. were cultured for in vivo testing in BioStat B-DCU fermenters (Sartorius; Göttingen Germany) using MRS broth (BD). Cultures were dried in LyoStar 3 lyophilizer (SP; Warminster, PA) and the lyophilized cake was powdered using sterile mortar and pestle. *Bacillus* spp. were cultured from single colonies in sporulation medium ([8 g Bacto nutrient broth, 1 g KCl, 0.12 g MgSO_4_·7H_2_O, 5 g dextrose]/L adjusted to pH 7.6 with NaOH, with 0.1% each 1 M CaCl_2_, 0.01 M MnSO_4_, and 1 mM FeSO_4_) for 96 h, followed by washing and resuspension in cold PBS (Invitrogen). Maltodextrin solution was added for a final concentration of 15%, and spores were spray dried in a Buchi mini spray dryer (Buchi, Flawil, Switzerland) at an outlet temperature of 104 °C. Dried spores with maltodextrin were mixed with 1.5% calcium phosphate as a desiccant.

The above probiotics were prepared into a premix suitable for aquaculture products based on colony forming units (CFU) from 3 to 8% lyophilized bacteria or 0.01–0.2% spores and food-grade excipients. For the freshwater study, five groups were tested for improvement in growth performance. For each test product, 10 kg pilot scale batches of commercial extruded feed pellets (Nutra Supreme HE 30, Skretting; protein, 49%; lipids, 23%; carbohydrates, 11.5%; fiber, 1%; ash, 8.5%; gross energy (MJ/Kg), 22.6) were top coated with 750 g of premix. Feed was coated first with premix, followed by 1% fish oil (100 g per 10 kg). 750 g of premix contained the desired CFUs of probiotic to achieve a final concentration of 2.23 × 10^6^ to 3.38 × 10^6^ CFUs/g of feed for *Bacillus* candidates and 1.0 × 10^8^ to 1.6 × 10^8^ CFUs/g of feed for *Latilactobacillus*. A similar process was used for generating TPs with larger feed pellets (Nutra Supreme HE 60, Skretting; protein, 49%; lipids, 24%; carbohydrates, 11%; fiber, 1.2%; ash, 8.0%; gross energy (MJ/Kg), 22.9) used for fish weighing above 60 g. After final drying, pellets were stored in plastic bags at 15 °C. For the saltwater study, three groups were tested for improvement in growth performance. For each TP, 10 kg pilot scale batches of commercial feed pellets with a size of 4 mm (Ewos® Micro 100) and/or 6 mm (Micro 250) were vacuum coated with 750 g of premix. More specifically, the premixes were suspended in an oil mix by using Ultra-Turrax® T50 (dispersion tool) and the feed pellets were then coated with the blended oil mix by a Forberg® rotating vacuum coater. After final drying, pellets were stored in plastic bags at 15–20 °C.

### Performance study in freshwater (7-week study)

A 7-week study was performed at Elanco Animal Health, Puerto Varas Aquarium Facility in Puerto Varas, Región de Los Lagos, Chile. Six hundred Atlantic salmon parr weighing 30–50 g were recruited from internal populations, distributed without intentional bias in twelve 100 L study tanks randomly allocated to 6 groups with 2 replicate tanks for each group. The fish were then acclimatized to the conditions of the feeding trial and fed with a basal commercial diet with composition appropriate to body weight for seven days without handling. The control group (negative control product, NCP) was fed commercial extruded basal diet and the probiotic groups were given TPs containing a combined dose of 2.23 × 10^6^ to 3.38 × 10^6^ CFUs/g of feed for *Bacillus* candidates and 1.0 × 10^8^ to 1.6 × 10^8^ CFUs/g of feed for *Latilactobacillus* candidates. Feed caliber was adjusted according to biweekly sample weights (20% of the fish per tank): Nutra Supreme HE 30 was delivered when fish weighed up to 60 g, and Nutra Supreme HE 60 when fish weighed more than 60 g. Fish were fed approximately 110% of the specific feed rate (SFR, ranged from 1.84 to 2.29) using a Skretting feed table manually or using an automatic feeder over an eight-hour period each day. The amount of feed delivered ranged from 0.037 to 0.153/kg/tank/day. Over the study period, fish were maintained in 100 L tanks of flow through fresh water under a photoperiod regime of 24-h day light. Water flow during the holding period and during experimental period was set at a rate to ensure a minimum of 2.0 total volume water exchange/hour. Supplemental oxygen was delivered as needed to the tank water to maintain appropriate saturated oxygen levels (70–130% saturation) and water temperature for all tanks was monitored daily.

### Performance study in saltwater (11-week study)

The goal of this study was to confirm the potential efficacy of *L. curvatus* strains PTA-127116 and PTA-127117 strains in saltwater with longer duration. Two additional *L. curvatus* candidates (LcELA388 and LsELA391) isolated from North American salmon intestine were also tested in this study. A 11-week study was performed at VESO (Chile). Four hundred fifty female Atlantic salmon parr weighing 125–145 g were recruited from Icelandic hatcheries (CIC), distributed without intentional bias into six 500 L study tanks randomly allocated to three groups with two replicate tanks for each group, and acclimatized to the conditions of the feeding trial and fed with a basal commercial diet with composition appropriate for body weight for fourteen days without handling. The control group (NCP-S) was fed commercial extruded basal diet and the probiotic groups were given TP1-S and TP2-S containing a combined dose of 6.05–6.29 × 10^7^ CFUs/g. Fish were fed approximately 110% of the specific feed rate (SFR, > 1.15) using a Skretting feed table using an automatic feeder. The amount of feed delivered ranged from 1.4 to 3.0/kg/tank/week. Over the study period, fish were maintained in 500 L tanks of flow through fresh water under a photoperiod regime of 24-h day light. Water flow during the holding period and during experimental period was set at a rate to ensure a minimum of 1.0–1.3 total volume water exchange/hour. Supplemental oxygen was delivered as needed to the tank water to maintain appropriate saturated oxygen levels (70–130% saturation) and water temperature for all tanks was monitored daily.

### Global untargeted metabolomic analysis

#### Study design

Global untargeted metabolomics was performed in the presence of various prebiotics and/or additives with MicroMGx (Chicago, IL). The selected prebiotics and/or additives with concentrations are listed in Table 2. To ensure statistical power, 3 biological replicates of each sample were analyzed. The total number of samples was 12 culture conditions × 2 different strains × 3 biological replicates = 72 total samples. Additionally, each of the culture media were extracted and analyzed to enable the identification and removal of background signals. Samples were analyzed in random sequence to manage batch effects.

**Table 2:** List of prebiotics and/or additives and their concentrations used in the study.

Study identifier	Condition name	Role	Description	Concentration
GLC	Glucose	Carbon source	Glucose	111 mM
MALT	Maltose	Carbon source	Maltose	58.5 mM
LAC	Lactose	Carbon source	Lactose	58.5 mM
FUC	Fucose	Carbon source	Fucose	30 mM
NAG	NAG	Simulant of fungal coculture	N-acetyl-glucosamine	20 mM
BS	Bile Salts	Microbiome metabolites	Bile-salts	0.3%
GOS	GOS	Prebiotic, carbon source	Galactooligosaccharide	0.5% w/v
IN	Inulin	Prebiotic, carbon source	Inulin (from chicory)	0.5% w/v
GOSC	GOS + vitC	Prebiotic, carbon source	Galactooligosaccharide and vitamin C	0.5% w/v + 2.84 mM
GOSD	GOS + vitD3	Prebiotic, carbon source	Galactooligosaccharide and vitamin D3	0.5% w/v + 0.021 mM
GOSZ	GOS + Zn	Prebiotic, carbon source	Galactooligosaccharide and zinc	0.5% w/v + 0.5 mM
GOSCMB	GOS combo	Prebiotic, carbon source	Galactooligosaccharide with vitamin C, vitamin D3, and zinc	0.5% w/v + 2.84 mM + 0.021 mM + 0.5 mM

#### Sample preparation

Cultures of each of the *Latilactobacillus* strains were grown overnight in MRS (BD) broth. Overnight cultures were then used to inoculate modified MRS broth (animal-origin peptones were replaced with vegetable proteose peptone; Sigma-Aldrich #29185) containing additives listed in Table 2. Cultures were grown anaerobically for 72 h. The cells and culture supernatants were separated by centrifugation for 5 min at 16,000×*g*. Culture supernatant was extracted using Oasis HLB solid phase extraction cartridges (Waters; Milford, MA) and then dried down in a vacuum centrifuge for later use.

### Metabolomics data acquisition

Samples were analyzed on a Q-Exactive mass-spectrometer (Thermo Fisher) coupled to an Agilent 1200-series UHPLC.

### Identification of metabolite features

Metabolite features are defined as a specific *m/z* signal associated with a specific retention time. The features shown in this report were determined to be significant because they showed a change in abundance across media conditions of greater than two-fold, with a significance between groups (one-way ANOVA, *P < 0.05). Where possible, metabolite features are assigned putative identifications by searching their observed accurate mass against a database of small molecules that are produced by bacteria.

### Association of putative metabolites with biomedical subject headings

Metabolite identities assigned to respective features were associated with medical subject headings (MeSH terms) following the method proposed by Sartor et al.^54^. Briefly, we evaluated the significance of the co-occurrence of metabolites and MeSH terms in the annotation of publications in PubMed (as of March 2020) using Fisher’s exact tests. Pairs of metabolites and MeSH terms with an FDR corrected *P*-value of 0.05 were considered significant. We only considered PubMed publications with both chemicals and MeSH terms annotated to them (7.8 million total publications), and only MeSH terms classified as “Descriptors” were considered for testing.

### Statistical analyses

Average weights of fish fed with different groups of probiotic-treated feed were compared using analysis of variance (ANOVA) for main effects and Dunnett’s test for multiple comparisons. Metabolite comparisons across media conditions were analyzed using ANOVA. A *P* < 0.05 was considered statistically significant.

### Ethis statement

This study was approved reviewed and approved by Elanco’s Animal Care and Use Committee (Institutional Animal Use and Care Committee; IACUC), reference ID EIAC-1471. Fish handling and sampling procedures were in compliance with Chilean legislation on the welfare of aquaculture animals, and this study was drafted in accordance with Chilean legislation of the welfare of animals used for scientific purposes.

## Results and discussion

### Isolation and identification of *Lactobacillus* spp. from healthy Atlantic salmon

With the goal to isolate and develop endogenous microbial isolates as potential probiotics to improve weight gain and enhance disease resistance in salmon, samples were collected from various growth stages (parr, smolts and grower) and major fish production sites (Norway, Chile, and North America)^1,55^. Parr and smolts were raised at 11–12 °C in freshwater while growers were raised at 8–12 °C in seawater.

A total of 900 microbial isolates were cultured from the intestines of Atlantic salmon (Tables 3 and 4). 16S rRNA sequencing identified 626 of these organisms (Supplementary File 2), which informed the selection of probiotic candidates from promising genera, sample diversity, and regulatory lists. 16S rRNA gene identification showed that the majority of the isolates belonged to the following genera: *Carnobacterium*, *Aliivibrio*, *Lactobacillus*, *Vibrio*, *Pseudomonas*, *Citrobacter*, *Photobacterium* and *Shewanella*. Consistent with the previous literature^56,57^, *Carnobacterium*, *Aliivibrio* and *Lactobacillus* were among the top probiotic genera isolated from Norwegian and North American samples. Carnobacteria are lactic acid bacteria which dominate fish hindgut by population^58^; and non-pathogenic strains of Carnobacteria have been previously shown to improve weight gain and disease resistance in farmed Atlantic cod and salmon^25,36,59^. Similarly, bathing with *Aliivibrio* strains improves growth and FCR, and reduces mortality in Atlantic salmon^60^.

**Table 3:** Description of probiotic library, *Bacillus* and *Lactobacillus* candidates.

Geographic Source	Sample type	Total isolates	*Bacillus*	*Bacillus* listed in QPS	*Lactobacillus*	*Lactobacillus* listed in QPS
Norway	Parr and grower intestine	268	0	0	8	8
North America	Grower intestine	394	0	0	10	10
Chile	Parr intestine	238	17	6	0	0

**Table 4:** *Bacillus* and *Lactobacillus* isolates and their growth profiles.

Strain	Geography	Sample	Water	Fish size (g)	Microaerophilic growth	Aerobic growth	15 °C growth
BvELA005	Chile	Intestine	Freshwater	38	ND	+	+
BvELA006	Chile	Intestine	Freshwater	38	ND	+	+
BvELA014	Chile	Intestine	Freshwater	95	ND	+	+
BsELA015	Chile	Intestine	Freshwater	95	ND	+	+
BsELA016	Chile	Intestine	Freshwater	95	ND	+	+
BsELA017	Chile	Intestine	Freshwater	95	ND	+	+
LcELA23	Norway	Intestine	Seawater	1500	+	+	+
LcELA29	Norway	Intestine	Seawater	1500	+	+	+
LcELA33	Norway	Intestine	Seawater	1500	+	+	+
LcELA92	Norway	Intestine	Seawater	1500	+	+	+
ATCC PTA-127116	Norway	Intestine	Seawater	1500	+	+	+
LcELA96	Norway	Intestine	Seawater	1500	+	+	+
LcELA98	Norway	Intestine	Seawater	1500	+	+	+
ATCC PTA-127117	Norway	Intestine	Seawater	1500	+	+	+
LcELA2	North America	Hindgut	Seawater	1200	+	+	+
LcELA59	North America	Foregut	Seawater	1200	+	+	+
LcELA60	North America	Foregut	Seawater	1200	+	+	+
LcELA61	North America	Foregut	Seawater	1200	+	+	+
LcELA62	North America	Foregut	Seawater	1200	+	+	+
LcELA64	North America	Foregut	Seawater	6000	+	+	+
LsELA65	North America	Foregut	Seawater	6000	+	+	+
LfELA68	North America	Foregut	Seawater	6000	+	+	+
LcELA388	North America	Foregut	Seawater	6000	+	+	+
LsELA391	North America	Foregut	Seawater	6000	+	+	+

Bv: *B. velezensis*, Bs: *B. subtilis*, Lc: *L. curvatus*, Ls: *L. sakei*, Lf: *L. fuchuensis.*

A total of 18 *Lactobacillus* isolates were cultured (Tables 3 and 4). Eight of these were isolated from the intestines of parr and grower salmon from Norway and 10 were isolated from the intestines of grower salmon from North America (Tables 3 and 4). Based on the 16S rRNA identification, the *Lactobacillus* isolates showed closest homology to *Latilactobacillus curvatus* (*L. curvatus*) and *Lactobacillus sakei* (*L. sakei*). Owing to their proven health benefits and long history of safe use, *Lactobacilli* are among one of the most commonly used probiotics in both human and animal health and are increasingly being evaluated as potential probiotics for fish^61^. With the goal to develop native *Lactobacilli* from salmon gut as Direct Fed Microbials (DFMs), we chose isolates belonging to *Lactobacillus* species from foregut and hindgut samples that are listed in QPS list put forth by the EFSA for further characterization^62^. Indeed, all *Lactobacillus* strains isolated from salmon intestine were identified by 16S rRNA sequencing as members of the QPS list. Despite the long-established potential for lactic acid bacterial probiotics, only one product containing *Pediococcus acidilactici* is commercially available^63^. *Latilactobacillus curvatus* (*L. curvatus*) and *Latilactobacillus sakei* (*L. sakei*) have been described for their use against *Listeria* in smoked salmon cold chain^64,65^, rather than probiotics in living fish. *L. sakei* was isolated and tested against *Aeromonas* in rainbow trout, a smaller freshwater fish^66^.

Seventeen spore-forming *Bacillus* strains were isolated from the intestines of Atlantic salmon parr from Elanco Animal Health, Inc. hatchery in Chile (Tables 3 and 4). Six of these *Bacilli* are listed in EFSA’s qualified presumption of safety (QPS) list, suggesting they may be considered safe for probiotic use^62^. Three of the *Bacillus* strains showed closest homology to *Bacillus velezensis* (*B. velezensis*) and three showed closest homology to *B. subtilis,* as identified by 16S sequencing and BLAST analyses. Spore-forming *Bacillus* are not a major part of the endogenous microbes of salmon^56,57^; in agreement with this, strains belonging to *Bacillus* were only isolated from Chilean salmon samples but not from Norwegian and North American salmon samples. The Chilean samples were sourced from freshwater and the Norwegian and North American samples were sourced from saltwater; the source of samples likely explains the selective isolation of *Bacillus* from Chilean salmon samples.

### Phenotypic characterization of *Lactobacillus* isolates

All the *Lactobacillus* strains were confirmed to be Gram positive by Gram staining. *L. curvatus* strains ATCC PTA-127116 and ATCC PTA-127117 were found to be motile, compatible with each other in an antagonistic assay and do not produce antibiotic like molecules. All strains had similar growth profiles. All the 18 strains grew on MRS agar and broth microaerobically and aerobically, at 15 °C and 23 °C (Table 4). This is consistent with their isolation from cold water fish in water temperature of 8.7–12 °C. *Bacillus* candidates also grew well at 15 °C (Table 4).

### In silico analyses of *Lactobacillus* isolates for safety and desirable probiotic properties

#### Genomic characterization

A total of 18 genomes were sequenced using Illumina platform. The genome properties, prediction, and annotation of different features are summarized in Table 5. To obtain nearly complete genomes, the genomes of *L. curvatus* strains ATCC PTA-127116 and ATCC PTA-127117, hereafter nicknamed as PTA-16 and PTA-17, were also sequenced by PacBio sequencing platform. PTA-16 contained 3 contigs yielding a total estimated genome size of 1.99 Mb and PTA-17 contained 2 contigs yielding a total estimated genome size of 1.97 Mb (Table 5).

**Table 5:** Genomic properties of *Latilactobacillus* strains.

Strain	Contigs	Depth of coverage	Genes	CDSs	Regulatory elements	Repeat regions	Ribosomal RNAs	Transfer RNAs	Transfer-messenger RNAs	Non-coding RNAs	Misc. binding	Misc. feature
ATCC PTA-127116	3	152	2,029	1,941	5	3	18	67	1	2	7	3
ATCC PTA-127117	2	746	2,007	1,921	6	1	18	65	1	2	7	3
LcELA388	2	347	2,133	2,047	6	4	18	65	1	2	8	3
LcELA391	3	413	2,135	2,045	7	1	21	66	1	2	6	3
LcELA23	78	801	1,885	1,835	5	3	3	44	1	2	7	3
LcELA29	88	689	1,922	1,867	6	1	3	49	1	2	7	3
LcELA2	79	364	1,913	1,863	6	1	3	44	1	2	7	3
LcELA23	94	794	1,897	1,842	5	3	3	49	1	2	7	3
LcELA59	71	300	1,770	1,721	6	2	3	44	1	2	7	3
LcELA60	67	347	1,778	1,728	6	2	3	44	1	2	7	3
LcELA61	66	304	1,872	1,820	6	2	3	46	1	2	7	3
LcELA62	69	304	1,809	1,761	6	1	2	44	1	2	7	3
LcELA92	79	598	1,879	1,829	5	3	3	44	1	2	7	3
LcELA96	79	731	1,890	1,840	5	3	3	44	1	2	7	3
LcELA98	76	877	1,914	1,865	6	1	2	44	1	2	7	3
LfELA68	45	337	1,842	1,816	6	1		23	1	2	6	3
LsELA64	41	229	2,089	2,032	7	3	2	52	1	2	6	3
LsELA65	22	419	1,916	1,883	7			30	1	2	8	3

#### Phylogenetic analysis

**Figure 1:**
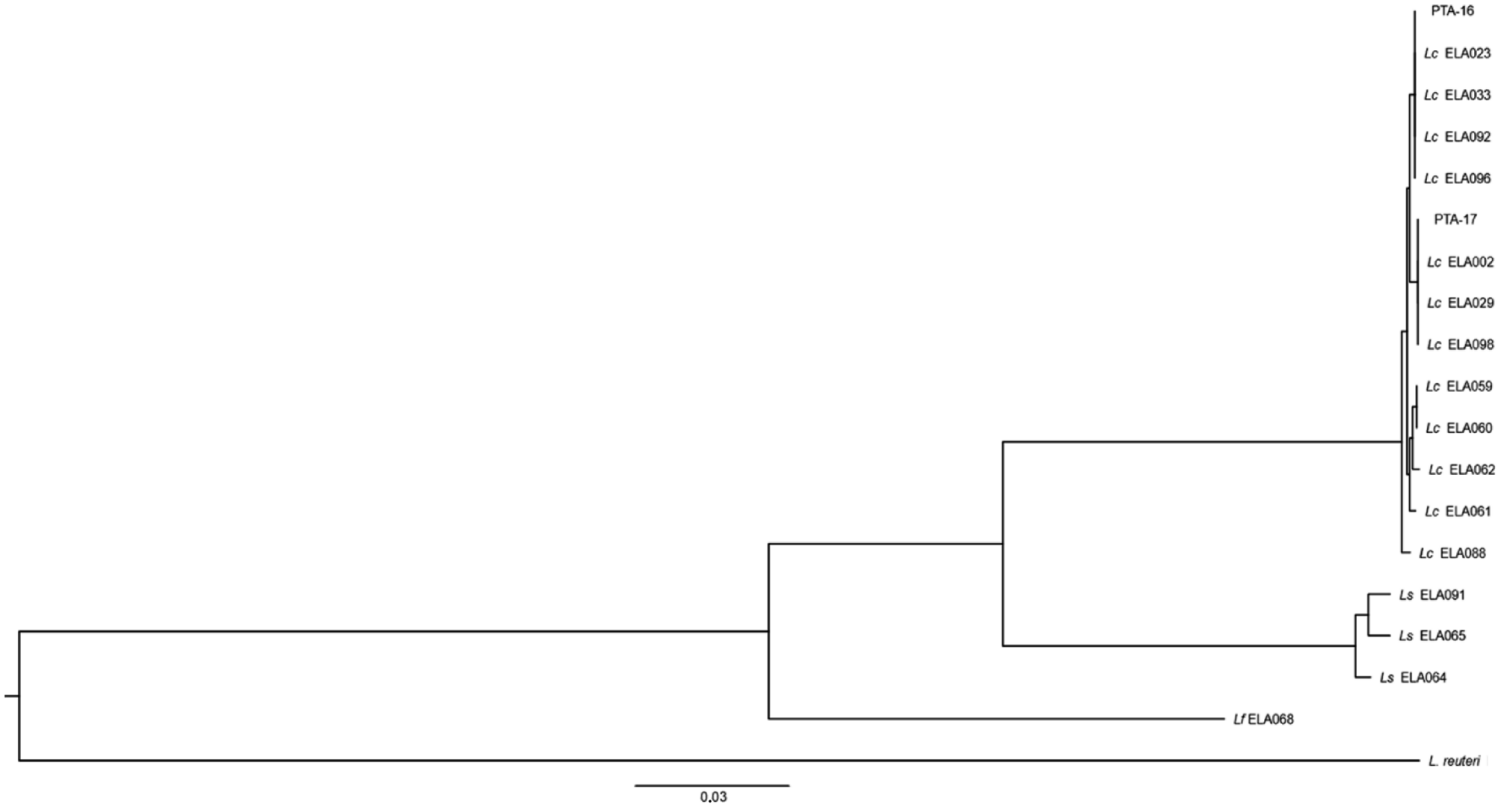
Phylogenetic relationship of *Latilactobacillus curvatus, Latilactobacillus sakei* and *Latilactobacillus fuchuensis* strains using 92 core genes. The phylogenetic relationship was explored using UBCG v3.0 and a maximum likelihood tree was inferred using GTR + CAT model. *L. reuteri* ATCC PTA-126788 was used as an outgroup.

Phylogenetic relationships of the genomes were explored with UBCG v3.0 using *L. reuteri* strain ATCC PTA-126788 as an outgroup. As shown in Fig. 1, different *Lactobacilli* neatly grouped into their respective species clades. Two of the *Lactobacillus* strains showed closest homology to *L. sakei,* 1 of the strain to *Latilactobacillus fuchuensis* (*L. fuchuensis*) and 15 of the strains, including PTA-16 and PTA-17, to *L. curvatus* (Fig. 1). Phylogenetic analysis has previously divided *L. curvatus* by its ability to metabolize plant-derived carbohydrates^67^; PTA-16 and PTA-17 were selected from diverse phylogenetic groups and fish specimens to form consortia for the in vivo study.

The genomes were further characterized for desirable safety and probiotic properties as described in Supplementary File 1 (Supplementary Tables 1–7 and Supplementary Fig. 1). Based on the comparative genomics analysis, PTA-16 and PTA-17 were selected for further in silico, in vitro and in vivo characterization. Comprehensive functional annotation of the *L. curvatus* strains PTA-16 and PTA-17 revealed presence of several putative genes important for probiotic efficacy (Supplementary Table 6 and 7) and contained no homologs of virulence and toxin genes. Probiotic bacteria are known to contain bioactive secondary metabolites that interact with other pathogenic bacteria to attenuate virulence^68–71^. Neither of the selected candidates seem to possess any bacteriocins found in Enzybase nor AntiSMASH. Analysis for antibiotic resistance genes revealed no hits using ResFinder, supporting PTA-16 and PTA-17 as safe probiotic candidates. Both PTA-16 and PTA-17 strains contained one coding sequence encoding L-lactate dehydrogenase (EC 1.1.1.27), which is responsible for lactic acid production. CDS encoding D-lactate dehydrogenase (EC 1.1.1.28) was not found in any of our strains. While the diversity of phages in gut ecosystems is getting increasingly well-characterized, knowledge is limited on how phages contribute to the evolution and ecology of their host bacteria^72,73^. Prophage analysis of PTA-16 and PTA-17 showed 7 prophage regions in each genome. Prophages can be advantageous for gut symbionts like *L. curvatus* by increasing its competitiveness in the intestinal niche^72^.

### Antimicrobial susceptibility of PTA-16 and PTA-17

**Figure 2:**
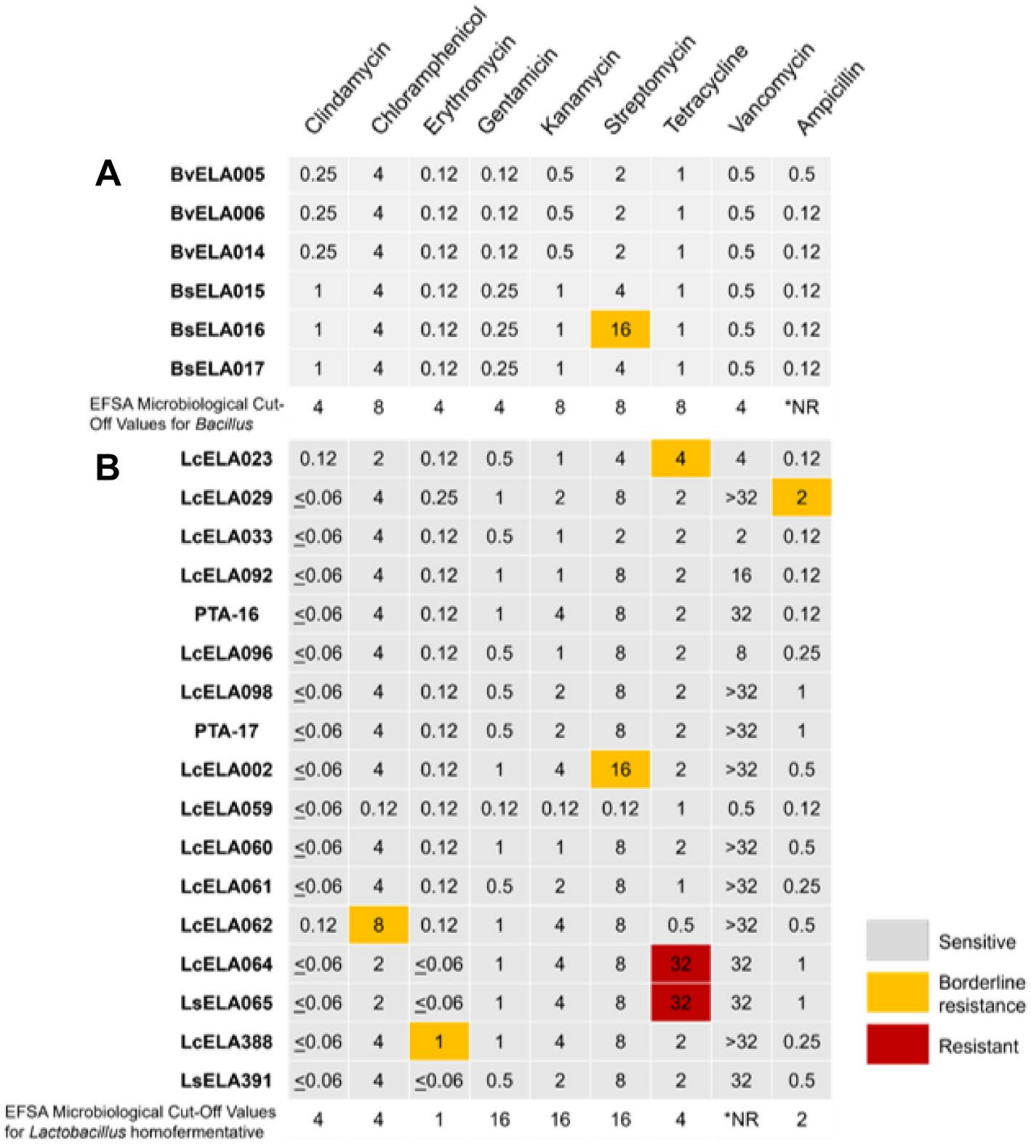
Antimicrobial susceptibility of *Bacillus* and *Lactobacillus* strains. MIC (μg/mL) values for each antibiotic tested against the respective genus are shown. Nine medically important antibiotics at a concentration range of 0.06–32 μg/mL were tested, and the respective antimicrobial susceptibility cut-off concentrations required for that genus are shown at the bottom of each panel. *NR = not required by EFSA. The results are representative of 3 independent experiments.

PTA-16 and PTA-17, along with other *Latilactobacillus* and *Bacillus* strains, were tested for antimicrobial susceptibility against relevant antibiotics, including ampicillin, vancomycin, gentamicin, kanamycin, streptomycin, erythromycin, clindamycin, tetracycline and chloramphenicol following EFSA guidelines^53^. PTA-16 and PTA-17 and other *Lactobacillus* and *Bacillus* strains LcELA33, LcELA92, LcELA96, LcELA98, LcELA59, LcELA60, LcELA61, LsELA391, BvELA005, BvELA006, BvELA014, BsELA015, and BsELA017 were sensitive to all relevant tested antibiotics^53^, with MIC values at or below the reported species characteristic cut-off values (Fig. 2). BsELA16 and LcELA2 were one- or two-fold dilutions above EFSA microbiological cutoff of streptomycin, and LcELA23, LcELA29, LcELA62, and LcELA388 were one- or two-fold dilutions above cut off values of tetracycline, ampicillin, chloramphenicol, and erythromycin, respectively (Fig. 2). This is considered acceptable due to the technical variation of the phenotypic method as recognized previously^74^. LfELA68 was not viable in any MIC medium tested; LsELA64 and LsELA065 were highly resistant to tetracycline (Fig. 2).

### Effect of in-feed administration of PTA-16 and PTA-17 (live) on growth performance in Atlantic salmon in freshwater

**Figure 3:**
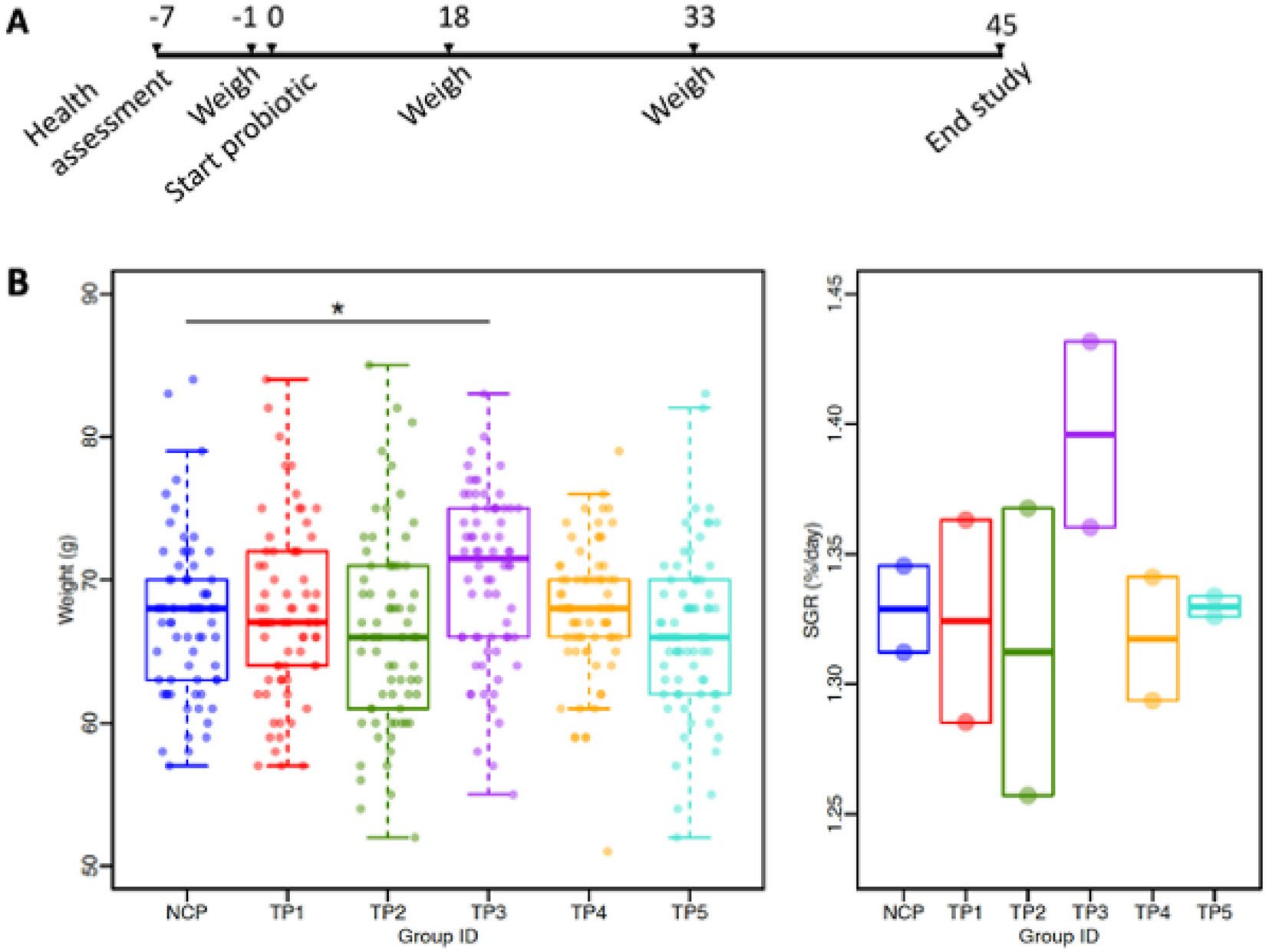
Effect of probiotic supplementation on the weights of salmon following daily administration in feed for 45 days in freshwater. (**A**) Timeline of experimental events. (**B**) Body weights and specific growth rates (SGR, %/day) for each group following daily administration of the respective probiotic candidates in feed for 45 days. Horizontal bar denotes mean. *P = 0.0172 for NCP vs TP3, ANOVA with Dunnett’s test (n = 70). TP, Test Product; NCP, negative control product.

The performance study was conducted at Elanco Animal Health, Puerto Varas Aquarium Facility in Puerto Varas, Región de Los Lagos, Chile. Five TPs and one NCP (control) were added to commercial fish pellets and fed to six groups of 100 fish each divided between two tanks per group. Ten fish were randomly selected from each tank, weighed, and returned on study days (SD) − 1, 18, and 33 (Fig. 3A). Seventy fish were weighed and euthanized at the end of the study on SD 45 (Fig. 3A). On study day 45, body weights were significantly different between groups (TP1 to TP5; *P* = 0.0007, n = 70); while TP1, TP2, TP4, and TP5 weights did not differ significantly from NCP, TP3 weighed significantly more (*P* = 0.0172, Dunnett’s test) (Fig. 3B). Average weights for TP1, TP2, TP3, TP4, TP5, and NCP respectively were 67.61 g, 62.40 g, 70.23 g, 67.79 g, 66.56 g, and 67.37 g (Fig. 3B). Weights relative to the control group were 0.4%, − 1.7%, 4.2%, 0.6%, and  − 1.7% for TP1, TP2, TP3, TP4, and TP5, respectively. There were no treatment-related gross pathologies (data not shown), mortalities or other safety concerns.

We hypothesized that native probiotics would be more effective than terrestrial probiotics due to their adaptation to fish physiology, and specific salinity and temperature requirements^26^. While *Bacillus* probiotics have shown promising growth improvement in salmonids^75^ and other fish^76^, our study revealed no improvement with terrestrial probiotics, nor with native *Bacillus* candidates, but showed potential indicators of efficacy only with native *Latilactobacillus* candidates.

### Effect of in-feed administration of PTA-16, PTA-17, LcELA388 and LsELA391 (live) on growth performance in Atlantic salmon in saltwater

**Figure 4:**
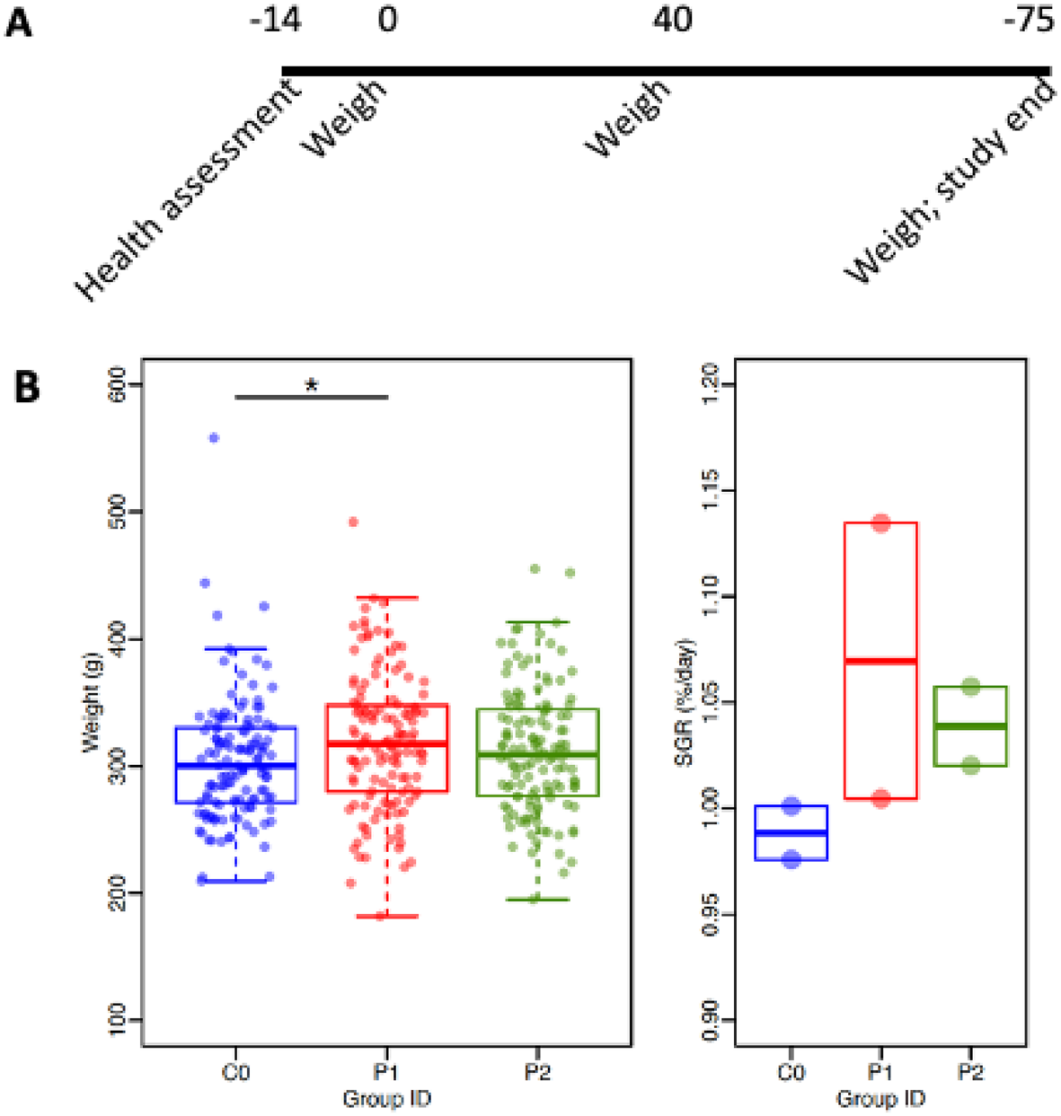
Effect of probiotic supplementation on the weights of salmon following daily administration in feed for 75 days in saltwater. (**A**) Timeline of experimental events. (**B**) Mean body weights ± standard error as well as specific growth rate (SGR, %/day) for each group following daily administration of the respective probiotic candidates in feed for 75 days. *P = 0.041 for Co vs P1, ANOVA with Dunnett’s test (n = 64). P1, Test Product 1-Saltwater; P2, Test Product 2-Saltwater; Co, Negative Control Product-Saltwater.

Two test products (TP1-S, TP2-S) and one negative control product (NCP-S) (Fig. 4) were added to commercial fish pellets and fed to three groups of 75 fish each divided between two tanks per group. All fish were weighed and returned on study days (SD) 0, 40, and 75. 20 fish were randomly selected from each tank, weighed, and returned on SD 18, 32, and 54. All fish were euthanized at the end of the study on SD 75 (Fig. 4). While TP2-S (Test Product 2) did not differ significantly from NCP-S, TP1-S weighed significantly more (*P* = 0.041, Dunnett’s test) (Fig. 4). Average weights for TP1-S, TP2-S, and NCP-S were 318.7 g, 311.8 g, and 304.5 g, respectively (Fig. 4). The specific growth rates (SGR) for TP1-S, TP2-S, and NCP-S were 2.23, 2.16 and 2.06, respectively. Weights relative to the control group were 4.7% and 2.4% for TP1-S and TP2-S, respectively. The 4.7% increase in final bodyweight for TP1-S translated to a 7.5% increase in average daily weight gain during the study compared to the control. There were no treatment-related gross pathologies (data not shown), mortalities or other safety concerns.

As diadromous fish, salmon live in both freshwater and seawater. *Lactobacillus* dominate the gut of saltwater salmon compared with freshwater fish^77^, and they are generally not recovered from very early stages^61^. Thus, it was not surprising that we recovered several isolates from saltwater fish but not freshwater fish. Interestingly, despite being isolated from seawater salmon, the *Lactobacillus* candidates PTA-16 and PTA-17 showed potential indicators of improved weight gain in salmon under both freshwater as well as saltwater conditions. Given the limited number of tanks and sample size as well as shorter duration of the study, the above potential indicators of efficacy need to be confirmed in larger and longer growth performance studies.

### Global untargeted metabolomics analyses of PTA-16 and PTA-17

**Figure 5:**
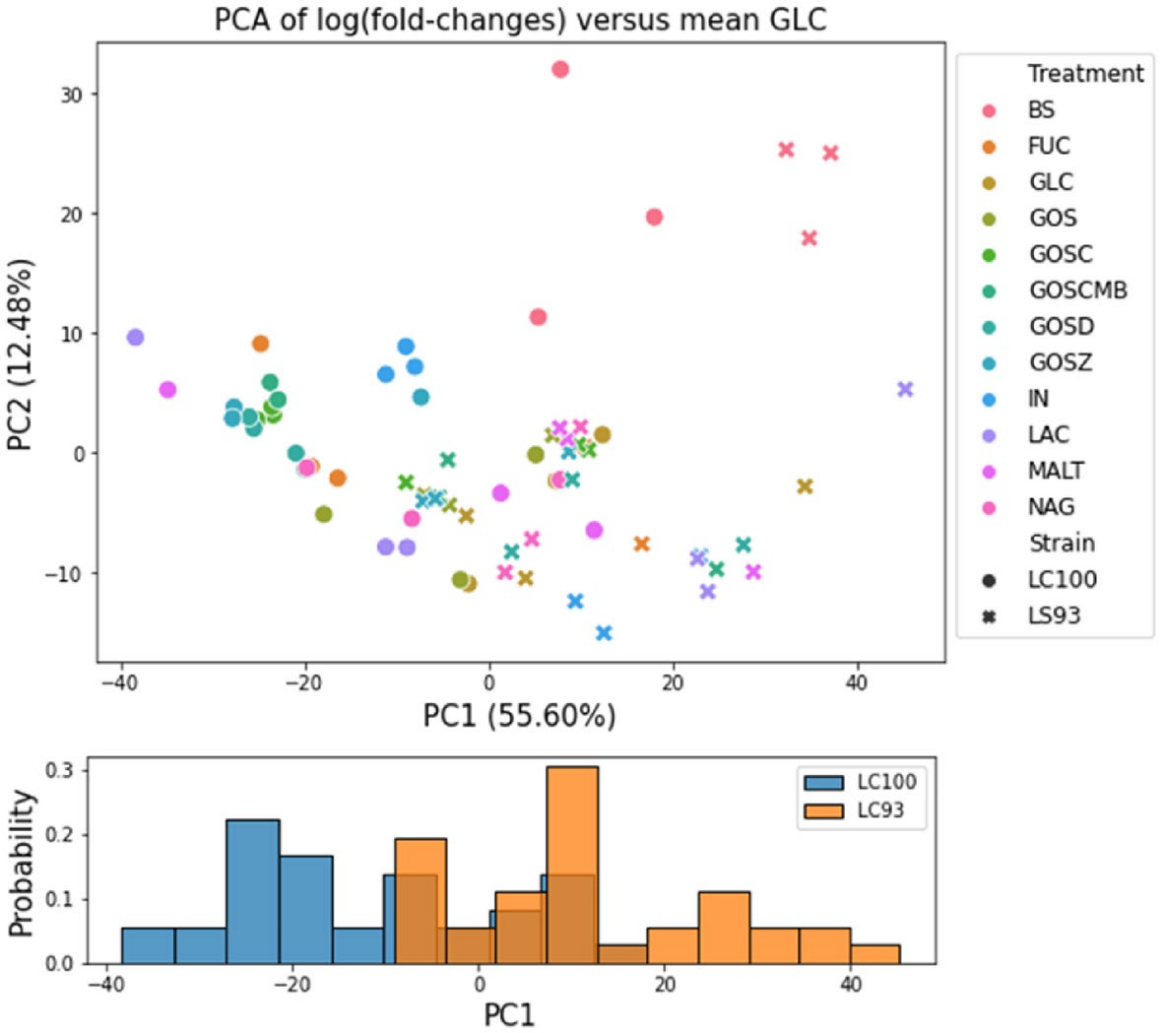
Principal component analysis (PCA) of feature abundance changes across media additives compared to media controls. Each marker in the figure represents one of three replicates in the corresponding treatment (shown in different colors). Numbers in parenthesis indicate the variance explained by each of the principal components. The histogram on the bottom represents the distribution of samples from each of the two strains along the first principal component. GLC, glucose. The data are representative of 3 independent experiments.

Based on this in vivo performance improvement, PTA-16 and PTA-17 were further analyzed for their ability to secrete various metabolites in the first comprehensive study in the presence of different prebiotics and/or additives. Synbiotics are the synergistic combination of prebiotic with probiotics, and since they have been shown to be beneficial in Caspian salmon^78^, we sought to identify potential prebiotics to enhance the efficacy of two *L. curvatus* candidates. In order to compare the responses of both strains to different prebiotics and/or additives, we compared their metabolomics profiles under different growth conditions. For the comparison, we carried out a principal component analysis (PCA) of the log_2_ fold-changes of feature abundances in each of the treatments compared to their average levels when the cells were grown on glucose. We only considered MS features present across all conditions with at minimum a twofold change in abundance compared to the glucose control in at least one sample (192 metabolites in total). As observed in Fig. 5, metabolomics profiles clustered by strain along the first principal component, representing over 50% of the variance in feature changes across strains, growth conditions and technical replicates. While media additives such as N-acetyl-glucosamine, and galactooligosaccharides (GOS) resulted in minor differences compared to the glucose control, additives including lactose, inulin, and GOS amended with vitamins and zinc resulted in larger metabolite shifts. Notably, the addition of bile salts resulted in distinct metabolomic profiles explaining most of the variance along the second principal component and clustering of the samples from both strains.

**Figure 6:**
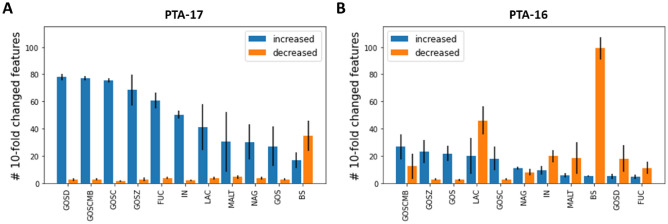
Features showing at least tenfold difference in abundance with different media additives compared to a control condition. (**A**) The number of features with higher than tenfold increase or decrease in abundance in media supplemented with different prebiotics and/or additives compared to a glucose media control for strain PTA-17. Error bars represent the standard error of the mean across replicates (n = 3). (**B**) Like (**A**), but for strain PTA-16. In both panels, prebiotics and/or additives are sorted according to the number of metabolites with increased abundance.

Comparing the magnitude of feature abundance changes showed that different prebiotics and/or additives caused more features to increase in abundance by more than tenfold in PTA-17 than PTA-16 when compared to a glucose control (Fig. 6). Many of the prebiotics and/or additives tested resulted in more than twice the number of features with increased expression in PTA-17. The opposite pattern, with more features decreasing in abundance by ten times or more across prebiotics and/or additives was observed in PTA-16 compared to PTA-17. Among the prebiotics and/or additives tested, lactose had a particularly strong effect reducing the expression of different features in PTA-16, whereas in both strains, bile salts reduced the levels of more features than any other prebiotics and/or additives. Our results indicate that both strains respond differently to prebiotics and/or additives supplementation in culture, with these molecules preferentially increasing metabolite levels in PTA-17 and decreasing them in PTA-16.

Looking at the abundances of individual features across prebiotics and/or additives instead of the changes relative to a control media showed less clustering of samples by strain (Supplementary File 1, Supplementary Fig. 2). While the overall composition of both strains for the features analyzed is similar across conditions, strains respond differently to distinct prebiotics and/or additives. Consistent with the above results, samples from both strains supplemented with bile acids cluster together and away from the remaining treatments. Additionally, addition of lactose led to highest divergence in metabolomics profiles between both strains.

Out of ~ 200 features analyzed, 136 could be mapped to 5 or less potential identities in the MicroMGX database and none were uniquely mapped (data not shown). In order to gain a broad idea of the possible physiological roles of these molecules, we followed the approach outlined by Sartor et al.^54^ to identify medical subject headings (MeSH terms) associated with metabolites detected in cultures of PTA-16 and PTA-17 based on their co-occurrence across published research. We identified at least one MeSH term associated with 39 out of 179 potential metabolite identities of MS features, representing 10,005 significant associations (FDR < 0.05) to 6239 MeSH terms (Supplementary File 1, Supplementary Fig. 3). These relationships illustrate the extent to which the identified compounds have been previously discussed in the scientific literature. Most of the associations uncovered were accounted for by adenine and biotin, whose central metabolic roles have been extensively studied. For the remaining metabolites, between 4 and 725 associations were identified.

Out of the recovered MeSH terms associated with potential metabolites produced by our strains, 46% corresponded to chemicals, 10% to diseases, 6% to physical processes and 5% to living organisms (Supplementary File 1, Supplementary Fig. 4). For example, multiple compounds (Aurafuron, Antramycin, Pladienolide, Epiderstatin, Eponemycin, Gancidin, and Medelamine) were associated with antibiosis, and metabolites including cycloleucine and 3-Methyleneindolenine were associated with body weight. Thus, while additional confirmation of the production of these molecules is necessary, our analysis provides a database to generate hypotheses about potential physiological impacts of the tested probiotics.

Metabolomics revealed that when 11 prebiotics and/or additives added to culture media, at least ten-fold PTA-16 and PTA-17 features were up- or down-regulated. This suggests that a synbiotic combination of our top probiotic candidates with one or more of these prebiotics is a promising approach to improve salmon performance. For PTA-17, features were especially increased in the presence of GOS supplemented with vitamin D, vitamin C, zinc, and all three combined. Vitamin C^79,80^ and zinc^81^ have already been studied for supplementation for Atlantic salmon health; their inclusion with fish feed would be accessible and familiar to farmers. GOS is a popular, widely available prebiotic. Features were especially decreased in the presence of bile salts, reflecting expected probiotic/digestive system interplay^82^. For PTA-16, features were especially decreased in the presence of lactose and bile salts. The differences in feature upregulation between species in the presence of GOS is predicted by previous work on *Lactobacillus* GOS metabolism^83^.

## Conclusions

In conclusion, we provide comprehensive genomic, phenotypic and metabolomic evidence to support the safety and indicators of potential efficacy of two novel *L. curvatus* probiotic candidates, PTA-16 and PTA-17 as potential probiotics for salmon. Our findings will inform future studies to further confirm and improve the potential efficacy of these two strains in larger studies and under different production and disease challenge conditions (salmon rickettsial septicemia). Future studies will also focus on exploring the effect of two *L. curvatus* candidates on feed and nutrient utilization, nutrient gain, immunity, hematological parameters, as well as product quality.

## Supplementary Information

Supplementary Information 1.

Supplementary Information 2.

## Data Availability

The datasets generated and/or analysed during the current study are available in the NCBI BioProject repository under the sample accession numbers SAMN21465945 to SAMN21465945 and SAMN23139425 to SAMN23139438. The data can be accessed using the following links: https://www.ncbi.nlm.nih.gov/bioproject/762592 and https://www.ncbi.nlm.nih.gov/bioproject/780402. The respective genome and bioproject accession numbers are also listed in Table 1.
