# Supplementary material for: In silico, in vitro and in vivo characterization of host-associated Latilactobacillus curvatus strains for potential probiotic applications in farmed Atlantic salmon (Salmo salar)
Source: Sci Rep. 2022 Nov 1;12:18417. doi: 10.1038/s41598-022-23009-y (PMC9626465; doi:10.1038/s41598-022-23009-y)
Supplement: Supplementary file 1 — Supplementary Information 1. [file 41598_2022_23009_MOESM1_ESM.docx]

**SUPPLEMENTARY FILE 1**

**1. Comparative genomics analyses**

Ortholog analysis was performed to identify paralogous and/or orthologous relationships between genomes of *L. curvatus, L. sakei* and *L. fuchuensis* strains. Genomic properties of *Latilactobacillus* strains using OrthoFinder are listed in **Supplementary Tables** **1 and 2**. Overall, 98.6% of the genes were shared between strains with 1215 orthogroups having membership of at least one gene in all 18 genomes. *L. curvatus* strains PTA-16 and PTA-17 shared 1681 and 1663 genes among them, respectively (**Supplementary** **Table 1**). Orthology-based multi-protein phylogenetic tree was used to identify optimal strain combinations from different clades (**Supplementary** **Figure 1**).

**2. Screening for prophages, ISs and transposases**

Genomes were scanned for the presence of mobile genetic elements such as prophages, ISs and transposases. Both PTA-16 and PTA-17 strains contained seven prophage regions each. However, there were 3 phage genes (all coding for Tyrosine recombinase protein) in both genomes that were outside of prophage regions (**Supplementary Table 3**). Putative IS and associated proteins predicted by ISEscan revealed 79 ORFs in 10 IS families in strain PTA-16 and 68 ORFs in 10 IS families in strain PTA-17 (**Supplementary Table 4**).

**3. Absence of virulence factors and toxins**

Both PTA-16 (3 contigs) and PTA-17 (2 contigs) strains were confirmed to be free of known virulence factors and/or toxins by comparing against virulence factor database (VFDB; search parameters of ³80% identity and ³80% alignment length/coverage), which is an integrated comprehensive online resource database for curating information about bacterial virulence factors and/or toxins (56). All other genomes were also free of virulence factors and toxins.

**4. Absence of acquired antimicrobial resistance genes**

The genomes of PTA-16 and PTA-17 along with other *Latilactobacillus* strains were searched for potential antimicrobial resistance genes against multiple AMR databases including NCBI-AMR, Resfinder DB and ARG-ANNOT using Abricate. The screening did not identify any potential antimicrobial resistance genes in any of the genomes except for LcELA65. LcELA65 contained a gene encoding tetracycline-resistant ribosomal protection protein (*tetW*) that confers resistance to tetracycline.

**5. Screening for genes involved in biogenic amines and toxins**

Functional annotation of the PTA-16 and PTA-17 genomes revealed that these strains do not contain any known protein-encoding genes involved in the production of biogenic amines with the exception of ornithine decarboxylase in the genome of PTA-16. Interestingly, both genomes had incomplete CDSs encoding tyrosine decarboxylase. No other toxins were identified in the genomes of both strains (**Supplementary Table 5**).

**6. Genes involved in the production of lactic acid and other beneficial metabolites**

Both PTA-16 and PTA-17 strains contained one CDS encoding for L-lactate dehydrogenase (EC 1.1.1.27). However, no CDS encoding for D-lactate dehydrogenase (EC 1.1.1.28) was found in any of the sequenced strains (**Supplementary Table 6**).

Several coding sequences involved in adhesion of Lactobacilli to intestinal epithelium including chaperonin GroEL, signal peptidase II and elongation factor Tu were identified in both PTA-16 and PTA-17 genomes (**Supplementary Table 7**). Search for desired stress tolerance features in PTA-16 and PTA-17 strains revealed the presence of three CDSs encoding for DNA protection during starvation. Another stress resistant gene putatively encoding for phosphate starvation-inducible PhoH-like protein was also found in both strains.

**Supplementary Tables**

**Supplementary Table 1.** Orthologs shared between Latilactobacillus strains.

|  | LcELA23 | LcELA29 | LcELA33 | LcELA92 | PTA-16 | LcELA96 | LcELA98 | PTA-17 | LcELA2 | LcELA59 | LcELA60 | LcELA61 | LcELA62 | LsELA64 | LsELA65 | LfELA68 | LcELA388 | LcELA391 |
| --- | --- | --- | --- | --- | --- | --- | --- | --- | --- | --- | --- | --- | --- | --- | --- | --- | --- | --- |
| LcELA23 | 0 | 1649 | 1786 | 1787 | 1775 | 1785 | 1648 | 1638 | 1646 | 1582 | 1581 | 1629 | 1604 | 1466 | 1444 | 1346 | 1640 | 1452 |
| LcELA29 | 1648 | - | 1652 | 1647 | 1651 | 1644 | 1825 | 1811 | 1832 | 1579 | 1577 | 1631 | 1584 | 1472 | 1460 | 1360 | 1639 | 1455 |
| LcELA33 | 1786 | 1654 | - | 1791 | 1783 | 1794 | 1654 | 1645 | 1653 | 1591 | 1593 | 1635 | 1606 | 1470 | 1444 | 1348 | 1653 | 1453 |
| LcELA92 | 1787 | 1650 | 1789 | - | 1777 | 1789 | 1647 | 1638 | 1645 | 1587 | 1586 | 1633 | 1607 | 1466 | 1443 | 1347 | 1643 | 1453 |
| PTA-16 | 1805 | 1681 | 1812 | 1815 | - | 1809 | 1676 | 1681 | 1678 | 1619 | 1609 | 1661 | 1636 | 1520 | 1493 | 1363 | 1677 | 1487 |
| LcELA96 | 1785 | 1650 | 1794 | 1790 | 1776 | - | 1647 | 1640 | 1647 | 1585 | 1585 | 1634 | 1604 | 1467 | 1444 | 1346 | 1645 | 1452 |
| LcELA98 | 1646 | 1825 | 1650 | 1643 | 1650 | 1641 | - | 1812 | 1826 | 1575 | 1573 | 1623 | 1583 | 1471 | 1459 | 1359 | 1639 | 1457 |
| PTA-17 | 1639 | 1826 | 1650 | 1648 | 1663 | 1643 | 1822 | - | 1823 | 1574 | 1569 | 1633 | 1589 | 1483 | 1472 | 1354 | 1656 | 1466 |
| LcELA2 | 1646 | 1833 | 1653 | 1644 | 1649 | 1643 | 1825 | 1811 | - | 1575 | 1576 | 1628 | 1580 | 1471 | 1460 | 1358 | 1640 | 1455 |
| LcELA59 | 1586 | 1580 | 1595 | 1590 | 1593 | 1586 | 1578 | 1572 | 1577 | - | 1696 | 1565 | 1599 | 1460 | 1430 | 1346 | 1578 | 1443 |
| LcELA60 | 1586 | 1580 | 1596 | 1590 | 1592 | 1587 | 1578 | 1572 | 1578 | 1697 | - | 1565 | 1600 | 1458 | 1428 | 1347 | 1579 | 1441 |
| LcELA61 | 1631 | 1639 | 1635 | 1633 | 1633 | 1635 | 1629 | 1629 | 1633 | 1570 | 1569 | - | 1581 | 1460 | 1439 | 1326 | 1628 | 1458 |
| LcELA62 | 1607 | 1594 | 1610 | 1613 | 1609 | 1608 | 1590 | 1586 | 1590 | 1602 | 1602 | 1583 | - | 1493 | 1462 | 1347 | 1589 | 1481 |
| LsELA64 | 1488 | 1498 | 1491 | 1490 | 1493 | 1489 | 1500 | 1495 | 1500 | 1485 | 1483 | 1487 | 1518 | - | 1737 | 1422 | 1540 | 1805 |
| LsELA65 | 1454 | 1467 | 1452 | 1454 | 1455 | 1453 | 1469 | 1465 | 1469 | 1444 | 1442 | 1451 | 1474 | 1715 | - | 1411 | 1471 | 1727 |
| LfELA68 | 1405 | 1429 | 1407 | 1406 | 1407 | 1405 | 1428 | 1423 | 1425 | 1412 | 1413 | 1390 | 1409 | 1471 | 1469 | - | 1408 | 1481 |
| LcELA388 | 1659 | 1686 | 1677 | 1679 | 1691 | 1670 | 1673 | 1672 | 1665 | 1597 | 1590 | 1653 | 1612 | 1550 | 1493 | 1354 | - | 1545 |
| LcELA391 | 1473 | 1487 | 1478 | 1480 | 1492 | 1476 | 1490 | 1485 | 1480 | 1466 | 1461 | 1487 | 1507 | 1808 | 1761 | 1424 | 1542 | - |

**Supplementary Table 2.** Summary of ortholog statistics of all *Latilactobacillus* strains.

|  | **LcELA23** | **LcELA29** | **LcELA33** | **LcELA92** | **PTA-16** | **LcELA96** | **LcELA98** | **PTA-17** | **LcELA2** | **LcELA59** | **LcELA60** | **LcELA61** | **LcELA62** | **LsELA64** | **LsELA65** | **LfELA68** | **LcELA388** | **LcELA391** |
| --- | --- | --- | --- | --- | --- | --- | --- | --- | --- | --- | --- | --- | --- | --- | --- | --- | --- | --- |
| **Number of genes** | 1791 | 1842 | 1808 | 1793 | 1867 | 1804 | 1833 | 1854 | 1842 | 1699 | 1700 | 1781 | 1743 | 2012 | 1839 | 1808 | 1965 | 1999 |
| **Number of genes in orthogroups** | 1791 | 1840 | 1808 | 1793 | 1865 | 1801 | 1833 | 1854 | 1842 | 1698 | 1699 | 1722 | 1717 | 1911 | 1802 | 1668 | 1923 | 1935 |
| **Number of unassigned genes** | 0 | 2 | 0 | 0 | 2 | 3 | 0 | 0 | 0 | 1 | 1 | 59 | 26 | 101 | 37 | 140 | 42 | 64 |
| **Percentage of genes in orthogroups** | 100 | 99.9 | 100 | 100 | 99.9 | 99.8 | 100 | 100 | 100 | 99.9 | 99.9 | 96.7 | 98.5 | 95 | 98 | 92.3 | 97.9 | 96.8 |
| **Percentage of unassigned genes** | 0 | 0.1 | 0 | 0 | 0.1 | 0.2 | 0 | 0 | 0 | 0.1 | 0.1 | 3.3 | 1.5 | 5 | 2 | 7.7 | 2.1 | 3.2 |
| **Number of orthogroups containing species** | 1768 | 1803 | 1777 | 1770 | 1764 | 1777 | 1801 | 1789 | 1806 | 1678 | 1677 | 1697 | 1682 | 1836 | 1769 | 1535 | 1791 | 1837 |
| **Percentage of orthogroups containing species** | 72 | 73.4 | 72.4 | 72.1 | 71.9 | 72.4 | 73.4 | 72.9 | 73.6 | 68.4 | 68.3 | 69.1 | 68.5 | 74.8 | 72.1 | 62.5 | 73 | 74.8 |
| **Number of species-specific orthogroups** | 0 | 0 | 0 | 0 | 0 | 0 | 0 | 0 | 0 | 0 | 0 | 0 | 2 | 3 | 0 | 16 | 14 | 11 |
| **Number of genes in species-specific orthogroups** | 0 | 0 | 0 | 0 | 0 | 0 | 0 | 0 | 0 | 0 | 0 | 0 | 5 | 6 | 0 | 36 | 28 | 23 |
| **Percentage of genes in species-specific orthogroups** | 0 | 0 | 0 | 0 | 0 | 0 | 0 | 0 | 0 | 0 | 0 | 0 | 0.3 | 0.3 | 0 | 2 | 1.4 | 1.2 |

**Supplementary Table 3.** Prophage regions in *L. curvatus* strains PTA-16 and PTA-17.

| **Chromosome** | **Feature** | **Start** | **End** |
| --- | --- | --- | --- |
| *L. curvatus* PTA-16 | |  |  |
| LCUR093_C2 | prophage_region | 181581 | 199968 |
| LCUR093_C2 | attL | 181565 | 181579 |
| LCUR093_C2 | attR | 197767 | 197781 |
| LCUR093_C2 | prophage_region | 268408 | 299250 |
| LCUR093_C2 | attL | 272006 | 272020 |
| LCUR093_C2 | attR | 298277 | 298291 |
| LCUR093_C2 | prophage_region | 853933 | 873317 |
| LCUR093_C2 | attL | 853917 | 853931 |
| LCUR093_C2 | attR | 870119 | 870133 |
| LCUR093_C2 | prophage_region | 912886 | 931408 |
| LCUR093_C2 | attL | 916688 | 916703 |
| LCUR093_C2 | attR | 930282 | 930297 |
| LCUR093_C2 | prophage_region | 974382 | 1003232 |
| LCUR093_C2 | attL | 977980 | 977994 |
| LCUR093_C2 | attR | 1004251 | 1004265 |
| LCUR093_C2 | prophage_region | 1057551 | 1070992 |
| LCUR093_C2 | attL | 1059650 | 1059665 |
| LCUR093_C2 | attR | 1068113 | 1068128 |
| LCUR093_C2 | prophage_region | 1435309 | 1457469 |
| LCUR093_C2 | attL | 1438014 | 1438029 |
| LCUR093_C2 | attR | 1458041 | 1458056 |
| *L. curvatus* PTA-17 | |  |  |
| LCUR100_C1 | prophage_region | 437719 | 457745 |
| LCUR100_C1 | attL | 437207 | 437219 |
| LCUR100_C1 | attR | 458540 | 458552 |
| LCUR100_C1 | prophage_region | 828138 | 835107 |
| LCUR100_C1 | attL | 831270 | 831284 |
| LCUR100_C1 | attR | 833078 | 833092 |
| LCUR100_C1 | prophage_region | 886098 | 914123 |
| LCUR100_C1 | attL | 889754 | 889766 |
| LCUR100_C1 | attR | 914250 | 914262 |
| LCUR100_C1 | prophage_region | 986251 | 1025100 |
| LCUR100_C1 | attL | 987383 | 987396 |
| LCUR100_C1 | attR | 1025491 | 1025504 |
| LCUR100_C1 | prophage_region | 1605278 | 1635616 |
| LCUR100_C1 | attL | 1604704 | 1604718 |
| LCUR100_C1 | attR | 1636559 | 1636573 |
| LCUR100_C1 | prophage_region | 1697109 | 1717945 |
| LCUR100_C1 | attL | 1695185 | 1695200 |
| LCUR100_C1 | attR | 1715272 | 1715287 |
| LCUR100_C1 | prophage_region | 1755737 | 1761501 |
| LCUR100_C1 | attL | 1753813 | 1753828 |
| LCUR100_C1 | attR | 1773900 | 1773915 |

**Supplementary Table 4**. IS elements identified in *L. curvatus* strains PTA-16 and PTA-17 genomes.

***L. curvatus* PTA-16**

| **Sequence identifier** | **Family name of IS element** | **Number of IS copies** | **% of genome sequence content spanned by IS elements** | **Length of sequence segments spanned by IS elements in a sequence** | **Length of specific sequence** |
| --- | --- | --- | --- | --- | --- |
| LCUR093_C1 | IS1380 | 1 | 6.12 | 1636 | 26736 |
| LCUR093_C2 | IS982 | 1 | 0.05 | 1007 | 1913219 |
| LCUR093_C2 | ISL3 | 1 | 0.08 | 1571 | 1913219 |
| LCUR093_C3 | IS5 | 1 | 1.84 | 886 | 48160 |
| LCUR093_C2 | IS110 | 2 | 0.18 | 3504 | 1913219 |
| LCUR093_C2 | IS21 | 2 | 0.27 | 5121 | 1913219 |
| LCUR093_C3 | IS30 | 5 | 12.52 | 6032 | 48160 |
| LCUR093_C2 | IS1380 | 6 | 0.51 | 9787 | 1913219 |
| LCUR093_C2 | IS3 | 14 | 0.92 | 17689 | 1913219 |
| LCUR093_C2 | IS30 | 46 | 2.5 | 47878 | 1913219 |
| ELA204093 | Total | 79 | 4.78 | 95111 | 1988115 |

***L.  curvatus* PTA-17**

| **Sequence identifier** | **Family name of IS element** | **Number of IS copies** | **% genome sequence content spanned by IS elements** | **Length of sequence segments spanned by IS elements in a sequence** | **Length of specific sequence** |
| --- | --- | --- | --- | --- | --- |
| LCUR100_C1 | IS256 | 1 | 0.05 | 996 | 1934542 |
| LCUR100_C1 | IS6 | 1 | 0.02 | 446 | 1934542 |
| LCUR100_C2 | IS6 | 1 | 6.01 | 2096 | 34896 |
| LCUR100_C2 | IS30 | 2 | 5.99 | 2092 | 34896 |
| LCUR100_C1 | IS21 | 3 | 0.29 | 5580 | 1934542 |
| LCUR100_C1 | IS5 | 3 | 0.26 | 5102 | 1934542 |
| LCUR100_C2 | IS3 | 3 | 5.55 | 1936 | 34896 |
| LCUR100_C1 | IS1380 | 5 | 0.44 | 8536 | 1934542 |
| LCUR100_C1 | IS3 | 19 | 1.18 | 22920 | 1934542 |
| LCUR100_C1 | IS30 | 30 | 1.73 | 33395 | 1934542 |
| ELA204100 | Total | 68 | 4.22 | 83099 | 1969438 |

**Supplementary Table 5.** Identified protein-coding genes putative for arginine deiminase pathway in *L. curvatus* PTA-16 and PTA-17.

| **Locus tag** | **Gene function** | **Start** | **End** | **Strand** | **Size (bp)** |
| --- | --- | --- | --- | --- | --- |
| *L. curvatus* PTA-16 | |  |  |  |  |
| K9847_05050 | Arginine repressor | 944607 | 945068 | Forward | 462 |
| K9847_05360 | Arginine repressor | 1002780 | 1003232 | Forward | 453 |
| K9847_08625 | Putrescine-ornithine Antiporter | 1653796 | 1655115 | Reverse | 1320 |
| K9847_08630 | ornithine decarboxylase (EC 4.1.1.17) | 1655189 | 1657345 | Reverse | 2157 |
| K9847_08960 | Arginine--tRNA ligase (EC 6.1.1.19) | 1724718 | 1726409 | Reverse | 1692 |
| K9847_05865* | Tyrosine decarboxylase | 1103609 | 1104739 | Forward | 1131 |
| *L. curvatus* PTA-17 | |  |  |  |  |
| LBW11_00900 | Arginine--tRNA ligase (EC 6.1.1.19) | 180044 | 181735 | Forward | 1692 |
| LBW11_04440 | Arginine repressor | 885565 | 886017 | Reverse | 453 |
| LBW11_04745 | Arginine repressor | 943482 | 943943 | Reverse | 462 |
| LBW11_03945** | Tyrosine decarboxylase | 790102 | 791968 | Reverse | 1867 |

**Note**: *internal stop; incomplete; partial in the middle of a contig; missing C-terminus; ** frameshifted; internal stop

**Supplementary Table 6.** Putative genes in L. curvatus strains PTA-16 and PTA-17 involved in lactic acid production.

| **Gene function** | **Strain** | **Chromosome** | **Position** | **Locus Tag** | **Size (bp)** | **Strand** |
| --- | --- | --- | --- | --- | --- | --- |
| L-lactate dehydrogenase (EC 1.1.1.27) | PTA-16 | LCUR093_C2 | 20800- 21777 | K9847_00245 | 978 | Forward |
|  | PTA-17 | LCUR100_C1 | 1917737- 1918714 | LBW11_09790 | 978 | Reverse |

**Supplementary Table 7.** Identified protein-encoding genes putative for adhesion by L. curvatus strains PTA-16 and PTA-17.

| **Locus Tag** | **Size** | | **Gene Function** | **Strand** |
| --- | --- | --- | --- | --- |
| *L. curvatus* PTA-16 | | |  |  |
| K9847_03715 | | 1626 | Chaperonin GroEL | Forward |
| K9847_05010 | | 1017 | Type I glyceraldehyde-3-phosphate dehydrogenase | Forward |
| K9847_05025 | | 1296 | Phosphopyruvate hydratase | Forward |
| K9847_06155 | | 1191 | Elongation factor Tu | Forward |
| K9847_06750 | | 456 | Signal peptidase II | Reverse |
| *L. curvatus* PTA-17 | | | |  |
| LBW11_03055 | | 456 | Signal peptidase II | Forward |
| LBW11_03645 | | 1191 | Elongation factor Tu | Reverse |
| LBW11_04775 | | 1296 | Phosphopyruvate hydratase | Reverse |
| LBW11_04790 | | 1017 | type I glyceraldehyde-3-phosphate Dehydrogenase | Reverse |
| LBW11_05950 | | 1626 | Chaperonin GroEL | Reverse |
| LBW11_03055 | | 456 | Signal peptidase II | Forward |

**Supplementary Figures**

**
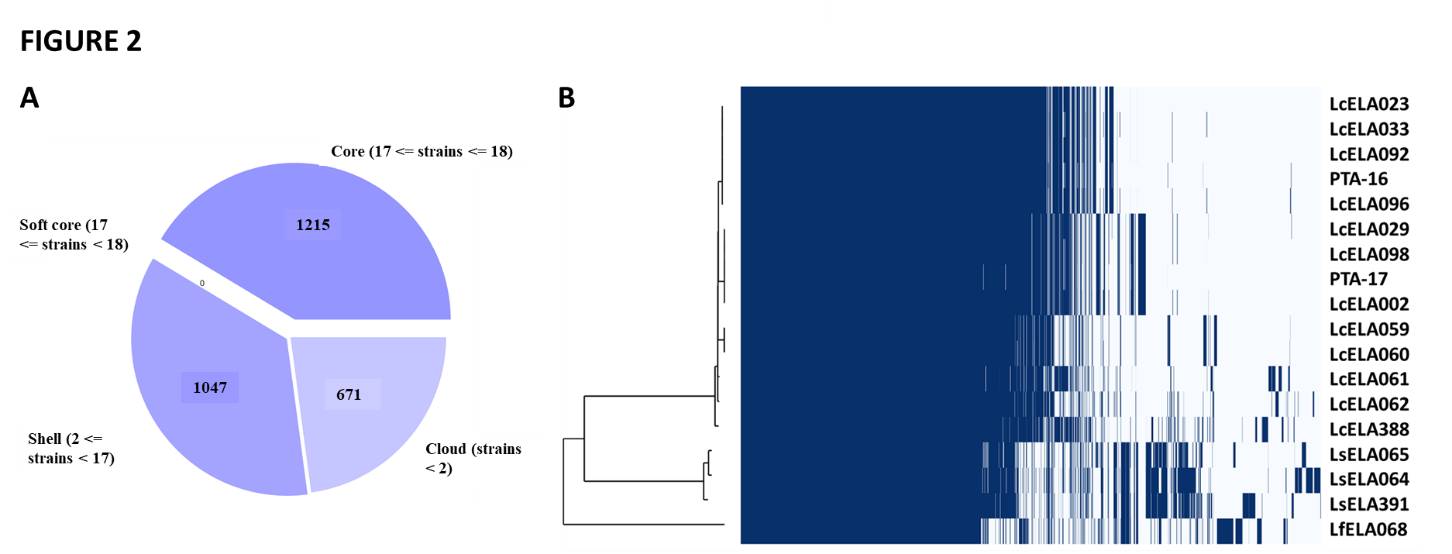
**

**Supplementary Figure 1**. **Pan-genome analyses of *L. curvatus*, *L. sakei* and *L. fuchuensis* strains as determined by Orthofinder.** **A**. The pan-genome pie chart showing gene content from core and accessory genes. **B**. A heatmap showing gene presence (dark blue) or absence (light blue) in each of the 18 *Latilactobacillus* strains. The core-genome tree generated was compared with a matrix where the core and accessory genes were either present or absent. Core, genes present in all strains; soft core, genes shared by the majority of the strains (>80%); shell, genes present in 15-80% of the strains; cloud, genes present in < 15% of the strains.

**
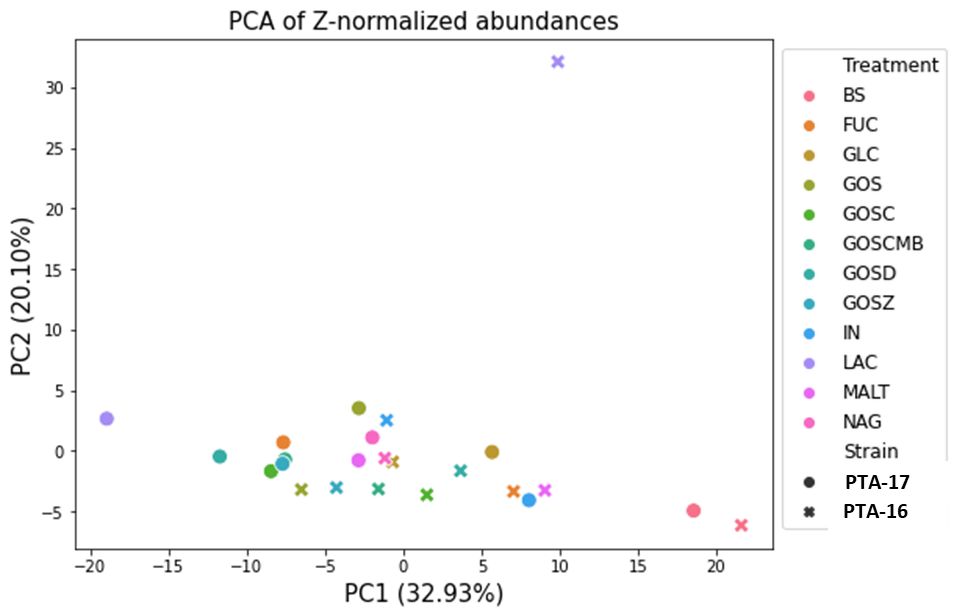
**

**Supplementary Figure 2. PCA plot (2-component) for standardized feature abundances in PTA-16 and PTA-17.** Each marker in the figure represents the mean of three replicates for each strain and growth condition. Abundance data for each feature was Z-score standardized before PCA analysis. Numbers in parenthesis indicate the variance explained by each of the principal components.

**
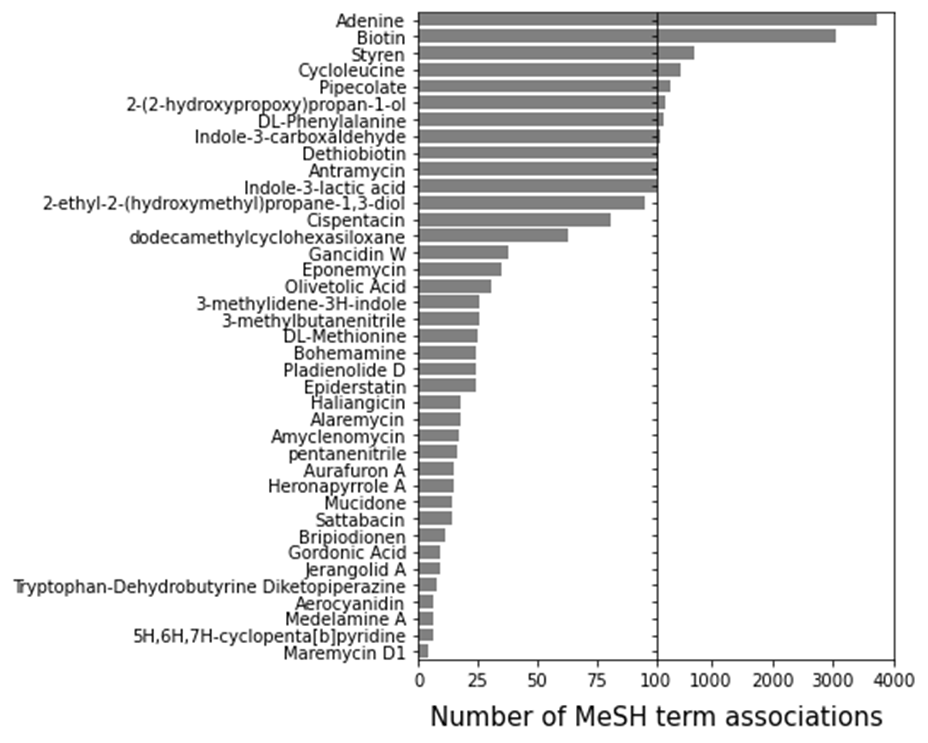
**

**Supplementary Figure 3. Number of MeSH terms associated with possible metabolites produced by PTA-16 and PTA-17.** MeSH terms were identified by their significant co-annotation with metabolites across PubMed articles.

**
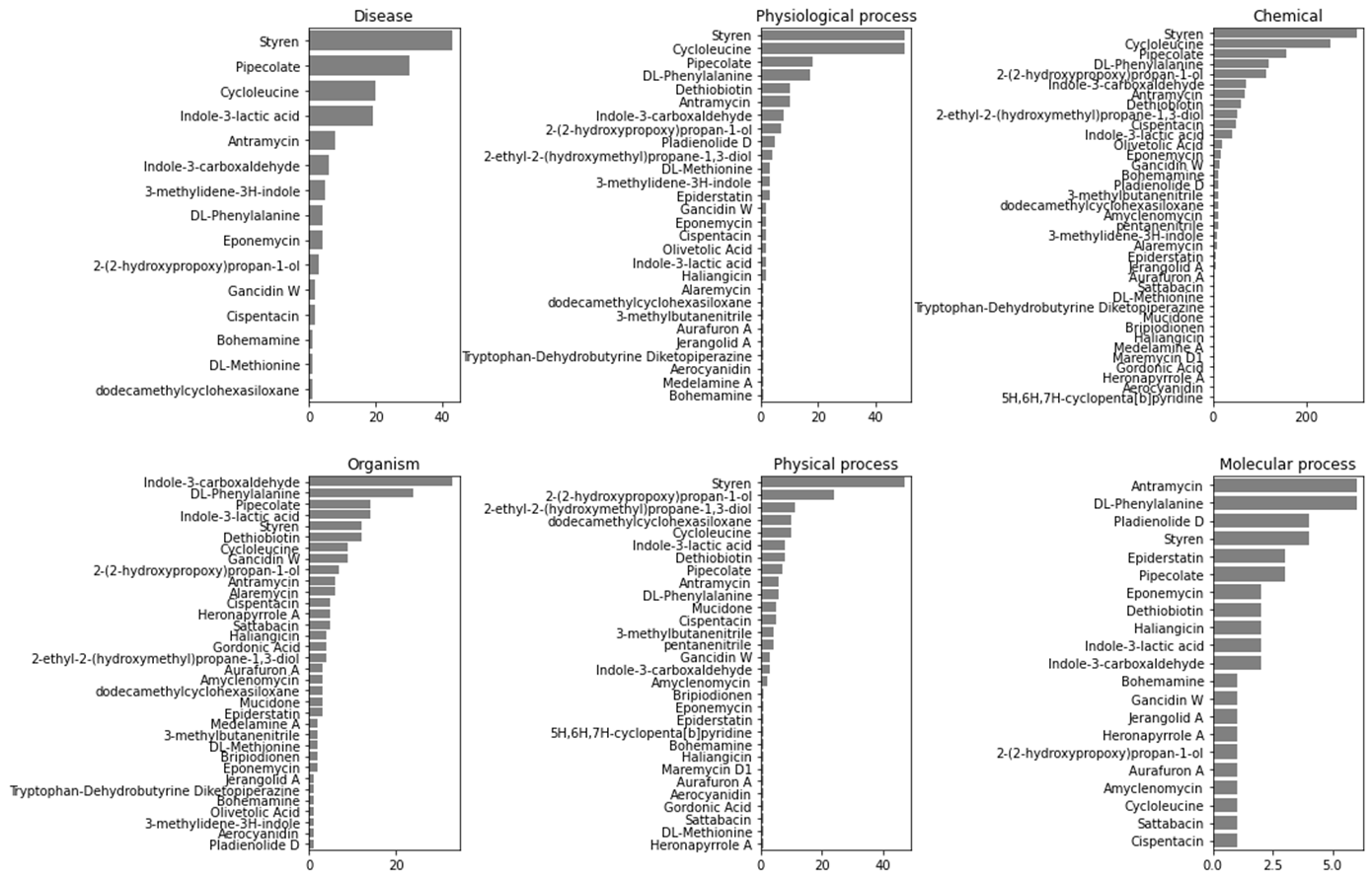
**

**Supplementary Figure 4. MeSH terms in different categories associated with potential compounds produced by PTA-16 and PTA-17.** MeSH terms associated *via* co-annotation in PubMed publications were classified based on the MeSH term ontology. Numbers for adenine and biotin were excluded.
